# Sex differences in behavioral measures of anxiety in a recessive gene knockout (*Pink1^–/–^*) rat model of Parkinson’s disease

**DOI:** 10.3389/fnbeh.2025.1646733

**Published:** 2025-09-02

**Authors:** S. M. Feehan, M. F. Kritzer

**Affiliations:** Department of Neurobiology and Behavior, Stony Brook University, Stony Brook, NY, United States

**Keywords:** non-motor deficits, prodrome, PTEN-induced putative kinase1, elevated plus maze, familial Parkinson disease

## Abstract

**Introduction:**

Parkinson’s disease (PD) is characterized by non-motor impairments including symptoms anxiety. These disturbances manifest in up to 40% of patients, most often early in the course of disease. While disruptive to all patients’ lives, signs of anxiety are also more prevalent and/or more severe in female PD patients. Unfortunately, anxiolytic drugs are rarely used to manage these signs, as these medications can increase PD patients’ risks for worsening of cognitive deficits and falls. The treatments commonly used in PD to improve patients’ motor function or lessen signs of depression are often without positive effect on measures of anxiety. Thus, clinical needs for successful treatment of anxiety symptoms in PD are frequently unmet.

**Methods:**

The work presented here used longitudinal Elevated Plus Maze (EPM) testing in male and female wild type rats and in male and female rats with knockout of the PTEN-induced putative kinase 1 gene (*Pink1^–/–^*) to determine whether these are suitable models for translational studies examining the neural substrates that underpin the sex-specific expression of anxiety symptoms in PD.

**Results:**

Behavioral testing in male and female wild type and *Pink1^–/–^* rats showed that *Pink1^–/–^* rats of both biological sex initially displayed hyperlocomotion and broad, possibly impulsive exploration of all portions of the elevated plus maze, including its open, unprotected spaces. While these behaviors persisted in *Pink1^–/–^* males, by 7 months of age, EPM performance in female *Pink1^–/–^* rats changed dramatically and included convergent behavioral measures indicative of significantly heightened anxiety, e.g., reduced open arm entries, slower speeds of ambulation in open arms, avoidance of distal ends of open arms. These and other signs of an anxiety remained through final testing of the female *Pink1^–/–^* cohort at 12 months of age.

**Discussion:**

Unlike a surprising number of other rodent models of PD that fail to emulate clinically observed anxiety and/or male/female differences in these signs, the data presented here identify *Pink1^–/–^* rats as strongly suited to lead translational efforts to better understand the neurobiological and neuroendocrine bases for anxiety symptoms in PD, their sex differences and their sex-specific sensitivities to therapeutic interventions.

## 1 Introduction

Parkinson’s disease (PD) is progressive neurodegenerative disorder widely recognized for adverse effects on patients’ motor function (Beitz, 2014). However, many patients diagnosed with PD also experience non-motor symptoms including anxiety disturbances (Chen and Marsh, 2014; Lintel et al., 2021; Pontone et al., 2009; Tan, 2012). These disturbances emerge early in the course of illness, are diagnosed in some 30 to 50% of PD patients and can take several forms including generalized anxiety, panic and phobias (Dissanayaka et al., 2014; Pontone et al., 2009; Wang et al., 2023). In all forms, however, anxiety disturbances in PD are often self-described as disabling and are well known to diminish patients’ quality of life and to increase care dependency and caregiver burden (Blundell et al., 2023; Geerlings et al., 2023; Hanna and Cronin-Golomb, 2012; Ray and Agarwal, 2020). It is thus all the more unfortunate that effective management of anxiety disturbance in PD is an area of ongoing clinical concern (Chen and Marsh, 2014). First, the use of anxiolytic and antidepressant medications that may be effective in other circumstances are often contraindicated in PD due to the potential for exacerbation of confusion and cognitive impairments and for increasing the likelihood of falls (Martinez-Ramirez et al., 2016; Weintraub, 2020). Further, although clinical trials focused on PD-related depression have shown that anxiety disturbances respond favorably to dopamine-or serotonin-targeting medications in some patients (Seppi et al., 2019; Troeung et al., 2013; Weintraub, 2020) for others these medications offer little to no symptom relief (Richard et al., 2012; Troeung et al., 2013; Weintraub, 2020). Moreover, because there have been no completed randomized controlled trials focused on treatments for anxiety in PD, these disturbances are frequently undertreated (Sawada et al., 2018). Thus, there exists significant need to better define the neural systems that underpin signs of anxiety specifically in contexts of PD and to develop better, safer ways to treat them. This in turn requires preclinical animal models that are validated for accessible, well-controlled study of the biological mechanisms of PD-related anxiety and expedited testing of emerging treatments. Thus, although data from brain imaging, EEG and other types of studies have made important inroads in identifying pathophysiological correlates (Carey et al., 2021; Dissanayaka et al., 2016; Perepezko et al., 2021; Swinnen et al., 2025; Yassine et al., 2024; Zhang P. et al., 2022), clinical studies of anxiety in PD are often challenged by patient and/or family reticence to acknowledge or discuss mental health concerns; by individual differences in the ways that patients experience anxiety; and by difficulties in distinguishing pathological anxiety from reactions to the stress of receiving a PD diagnosis (Gallagher et al., 2010; Khatri et al., 2020). Further, while the signs and symptoms of anxiety disturbance in PD are more common and more severe in female patients (Cattaneo and Pagonabarraga, 2025; Couture et al., 2024; Nicoletti et al., 2017), because PD is more prevalent overall in males overall (Cerri et al., 2019; Patel and Kompoliti, 2023), there are fewer female patients diagnosed with PD available for study. These and other challenges to clinical studies are mitigated in studies using preclinical animal models where population variance can be reduces, where subjective scales can be replaces with objective measures of stress and anxiety and where studies in female subjects can be adequately powered.

Animal and especially rodent models have been successfully used to investigate non-motor symptoms of anxiety in PD (Faivre et al., 2019; Hayley et al., 2023; Titova et al., 2017). The majority of these studies have employed selective neurochemical dopamine lesions, environmental toxin exposures or α-synuclein overexpression to model early, pre-motor stages of PD– and in most increased behavioral measures of anxiety have been observed (Boi and Fisone, 2024; Bustelli et al., 2024; Campos et al., 2013; Decourt et al., 2021; Faivre et al., 2019; Prediger et al., 2012; Taylor et al., 2010). To date, however, studies have mainly been carried out in male subjects alone and thus offer little to no information about face validity for sex differences in anxiety disturbances in PD in several of these models. Further, studies in which both sexes were examined, e.g., those using α-synuclein over-expressing mice, found greater indices of anxiety in males, which is the opposite of what is observed for anxiety in PD clinically (Lamontagne-Proulx et al., 2023). Among genetic rat and mouse of PD, studies using novel open field, elevated plus maze and other behavioral tests have uncovered increased measures of anxiety in some strains, diminished anxiety in others, and in nearly all cases, information about sex differences is unavailable (Boi and Fisone, 2024; Decourt et al., 2021; Faivre et al., 2019; Zhang T. D. et al., 2022). In sum, rodent models of PD that recapitulate clinical features of both increased anxiety and enhanced vulnerability to anxiety disturbances among female subjects are largely lacking. The studies presented explored whether rats with knockout of PTEN-induced putative kinase 1 gene (*Pink1^–/–^*) might serve as sex-specific preclinical models of anxiety in PD that are suitable to fill this gap.

Recessively inherited loss of function *PINK1* mutations are the second most common mutation in autosomal recessive forms of PD and are causally linked to early onset, familial forms of disease (Kumazawa et al., 2008; Scarffe et al., 2014; Valente et al., 2004). While the numbers of PD cases involving *PINK1* are small, recent demographic data identify several global “hot spots” for *PINK1*-related PD where prevalence values exceed quantitative definitions of rare illness (Yin and Dieriks, 2025). Further, PD cases attributed to *PINK1* are known to share core features with idiopathic PD, including progressive dysregulation and neurodegeneration in key neurotransmitter systems, abnormal α-synuclein accumulation and progressive motor deficits (Gonçalves and Morais, 2021; Kasten et al., 2018; Ryan et al., 2015). Particularly relevant to the present studies are findings that patients with *PINK1*-related forms of PD are also at elevated risk for non-motor deficits impacting cognition and neuropsychiatric domains including anxiety (Kalinderi et al., 2024). Thus, it is not surprising that rat and mouse strains engineered to carry loss of function or knockouts of *Pink1* not only recapitulate motor deficits of PD (Dave et al., 2014; Lamberty et al., 2023; Soto et al., 2024a) including those involving orofacial movements and vocalization (Grant et al., 2015; Hoffmeister et al., 2021; Johnson et al., 2020; Kelm-Nelson and Gammie, 2020; Kelm-Nelson et al., 2018; Marquis et al., 2020), but also show non-motor impairments in cognition and memory (Desai et al., 2025; Desai et al., 2025; Pinizzotto et al., 2022; Soto et al., 2024b).

Studies in *Pink1* deficient mice have also identified links between dysregulation of mitophagy, cellular stress responses and anxiety (Agnihotri et al., 2019; Duan et al., 2021; Li et al., 2024) and several studies in *Pink1^–/–^*rats have identified gene impacts on behavioral measures of anxiety in open field, light/dark box and elevated plus maze testing (Cai et al., 2019; Hoffmeister et al., 2022; Lechner et al., 2022; Marquis et al., 2020). However, inconsistencies across studies have left it unclear whether and to what extent *Pink1^–/–^* rats aptly recapitulate the female over male differences in PD-related disturbances in anxiety that are observed clinically. Based in part on recent evidence showing that *Pink1^–/–^* rats model the increased vulnerability of male PD patients for non-motor deficits in cognition and memory (Desai et al., 2025; Desai et al., 2025; Kelm-Nelson et al., 2021), it was hypothesized that this strain would also recapitulate the greater vulnerability of female PD patients to anxiety disturbances. Thus, behavioral indices of heightened anxiety were expected to emerge in early adulthood to greater to exclusive extents in female compared to male Pink1 rats, and to progressively worsen over time. These predictions were tested in longitudinal (repeated) elevated plus maze (EPM) behavioral testing in male and female wild type (WT) and *Pink1^–/–^* rats from 3 through 9 or 12 months of age that employed standard analyses of well-validated indices of anxiety and additional maze compartment- and sub compartment-specific assessments to corroborate principal findings. For all measures, sex differences were evaluated in WT male and female rats and sex-specific effects of the *Pink1^–/–^* genotype on EPM behaviors were evaluated by comparing data from male and female *Pink1^–/–^* to sex- and age-matched WT controls. Estrous cycles were also tracked to determine regularity of cycling in WT and *Pink1^–/–^* female rats. However, because the of numbers of WT and/or *Pink1^–/–^* female rats that were in estrous cycle stages characterized by high (estrus or protestrus) vs. low (diestrus I or II) circulating hormone levels on testing days turned out to be strongly skewed, planned comparisons of data stratified by estrous cycle stage were dropped from the analyses.

## 2 Materials and methods

### 2.1 Animal subjects

All procedures involving animals were approved by the Institutional Animal Care and Use Committee at Stony Brook University and were performed in accordance with the U.S. Public Health Service Guide for Care and Use of Laboratory Animals to minimize their discomfort.

Animal subjects were male and female Long Evans rats that were either wild type (WT) or *Pink1* knockouts [*Pink1*^–/–^ (LE-Pink1^*em*1*Sage*^^–/–^)]. All rats were purchased at 6–7 weeks of age (Envigo, Madison, WI, USA) and were double housed by sex and genotype for the duration of the study.

The male rats (8 WT, 16 *Pink1^–/–^*) served as subjects in a previous study that included some EPM data (Pinizzotto et al., 2022). However, the data presented here are either analyzed for the first time (7-months time point) or re-analyzed using different, fully automated methods (3- and 9-months time points). The female rats (10 WT, 12 *Pink1^–/–^*) were tested approximately 1 year after the males.

Rats were maintained under a 12-h non-reversed light-dark cycle (standard translucent tub cages, Lab Products, Inc., Seaford, DE, USA). Each cage contained enrichment objects (Nyla Bones, Nylabone, Neptune, NJ, USA) and ground corn cob bedding (Bed O’ Cobs, The Anderson Inc., Maumee, Ohio, USA). Food (Purina PMI Lab Diet: ProLab RMH 3000) and water were available ad libitum.

### 2.2 Weight and estrous cycle monitoring

Rats were weighed not less than every other month to ensure continued good health. Beginning 1 week after their arrival, female rats were vaginally lavaged with saline daily for 2 weeks. Thereafter, lavages were performed every 2–3 days. At ∼6 months of age, visual inspection of the vaginal opening replaced lavage as a less stressful method of identifying estrous cycle phases (Ekambaram et al., 2017). Visual inspections were performed on behavioral testing days and intermittently in between.

### 2.3 Behavioral testing

Testing took place in a rat behavioral core facility. A central room in the suite was used to hold rats in home cages for habituation and prior to being transported into an adjacent 10–12 ft square sound attenuated testing room where the plus maze was kept. This testing room had adjustable high contrast spatial cues on the walls and overhead digital cameras to archive trials. The room cues were changed for each time point evaluated.

Rats were tested during subjective nights between the hours of 9:00am and 1:00pm under ambient white lighting (∼ 260 lux). In addition to the Elevated Plus Maze paradigm, all rats in this study were tested on multiple object recognition-based memory tasks and tests of motor function on bi-monthly bases. However, plus maze testing was always conducted first.

**FIGURE 1:**
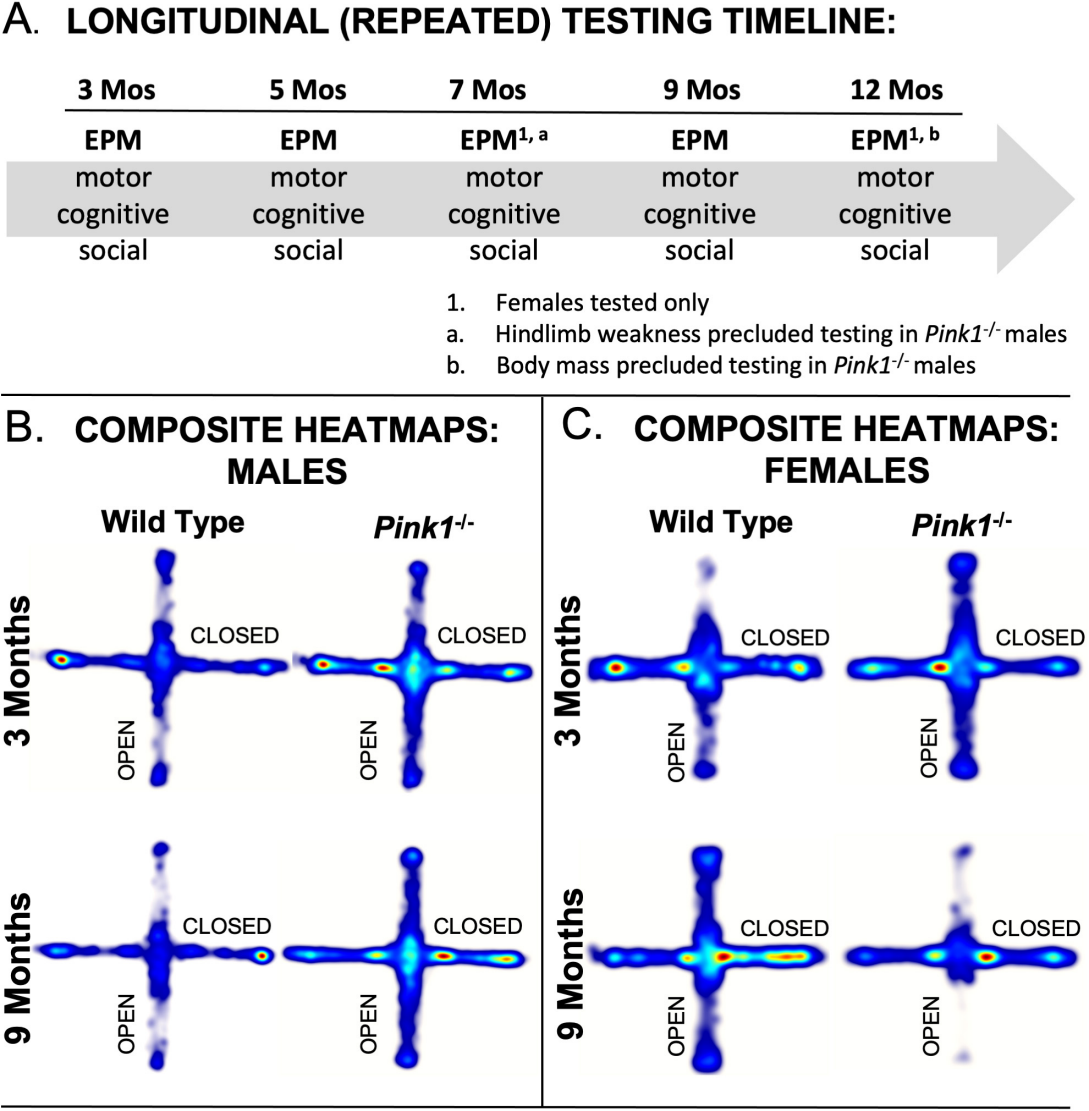
**(A)** Schematic showing the timeline of Elevated Plus Maze (EPM) testing noting the other types of tests rats were subjected to and the ages in months (Mos) where only female subjects were evaluated on EPM. Composite heat maps providing visual representations of the times that wild type and *Pink1^–/–^* male rats **(B)** and that wild type and *Pink1^–/–^* female rats **(C)** spent in different parts of the elevated plus maze during initial testing at 3 months of age and during testing at 9 months of age (the oldest age at which male rats were tested. Warmer colors identify zones where rats in each group spent the most time, and whitish zones are where the group spent minimal time. Wild type male rats showed more exploration of open arms during testing at 3 compared to 9 months of age, whereas wild type females initially avoided open arms, but explored these zones more and more with repeated testing. Performance in male *Pink1^–/–^* rats was similar at 3 and 9 months; at both times, these rats explored more overall and spent relatively more time in open arms and the center platform compared to wild type controls. Female *Pink1^–/–^* rats also initially explore open arms more so than wild type female controls. However, by the end of testing, the rats in this group spent minimal time in these open portions of the plus maze.

Female rats were tested at 3, 5, 7, 9 and 12 months of age. Male rats were tested at 3, 5 and 9 months old. The males were not tested at 7 months of age due to significant hindlimb weakness noted in more than half of the *Pink1^–/–^* males, which proved to be transient; rats tested at 9 months of age showed no obvious motor deficits. At 12 months old, however, it was not deemed safe to test the WT or *Pink1^–/–^* males due to their large sizes. Figure 1A shows a schematic for the timeline of behavioral testing. During intervals when rats were not behaviorally tested, they spent roughly 1 h per week in groups of 2–6 in a large, dimly lit 6 ft square enclosures that contained tunnels, platforms and other larger scale objects for them to interact with.

### 2.4 Apparatus

The elevated plus maze used was constructed of pressed white laminate. The plus configuration of the maze was formed by: two opposing closed arms (14 cm wide, 52 cm long) that were enclosed on three sides by walls that were 28.5 cm tall; two opposing open arms (14 cm wide, 52 cm long); and an open central platform in between the four arms that measured 14 cm square. The maze was supported on 36 in legs, the floor beneath the maze was covered in 4 in thick foam padding and a digital camera (webcam) was suspended 51 cm above the center of the maze.

### 2.5 Elevated plus maze testing

To initiate trials, rats were brought from the central holding room into the testing room in clean transport cages and were immediately and gently placed on the central platform of the maze facing away from the handler. The handler then exited the room and rats were given 5 min to freely explore the maze. All maze surfaces were cleaned with a 70% ethanol solution before and after each trial.

### 2.6 Data analysis

#### 2.6.1 Estrous cycle determination

Vaginal cytology samples were evaluated using light microscopy and differential interference contrast illumination. Estrous cycle stages were cytologically identified by relative abundance of nucleated epithelial cells (proestrus), cornified and anucleated epithelial cells (estrus) and leukocytes (diestrus) in the samples. Evaluations estrous cycle stage using visual inspections of the vaginal opening were based on the width of the vaginal opening (wide to gaping = estrus or proestrus; narrow to closed = diestrus) and the coloration of surrounding tissue (pink = estrus or proestrus; blueish = diestrus)

#### 2.6.2 Behavioral data

All data were evaluated from overhead digital recordings of the trials. Digital tracking of animals’ center points (Noldus Ethovision XT) was used to measure exploration in the maze as a whole, with respect to defined maze compartments, i.e., center platform, open and closed arms, and within sub compartments of maze arms, i.e., proximal, medial and distal thirds. These measurements and their units are listed in Table 1. Because time-dependent measurements made within proximal, middle and distal subdivisions of maze arms are influenced by the total amounts of time rats are in these zones, these values were measured and compared as percentages of total times spent in open or closed arm compartments.

**TABLE 1:** List, definition, and basic interpretations for behaviors evaluated in the elevated plus maze testing.

Behavioral variable	Definition	Behavioral interpretation
Times spent in closed arms: Total Proximal ends Distal ends	Cumulative time spent within a defined maze compartment.	Total: increased time associated with higher anxiety. Distal: increased time indicates strong anxiety response. Proximal: increased time associated with decision making, risk assessment with avoidance > approach.
Times spent in open arms: Total Proximal ends Distal ends	Cumulative time spent within a defined maze compartment.	Total: increased time associated with lower anxiety. Distal: increased time associated with lower/lowest anxiety. Proximal: increased time associated with higher anxiety, less exploration.
Times spent in center: Total	Cumulative time spent within a defined maze compartment.	Total: increased time associated with decision making, risk assessment with approach > avoidance.
Center, closed or open arm entries	Directional entries into maze space, minimum depth of one body length	Greater numbers associated with greater interest in exploration, compartment specific interpretations as above.
Total time spent ambulating	Cumulative time spent moving faster than 1.75 cm/s	Motor function, compartment specific interpretations as above.
Distance traveled	Total length of tracked pathways/path lengths	Motor function, compartment specific interpretations as above
Average speed of ambulation	Average of velocities greater than 1.75 cm/s	Motor function, compartment specific interpretations as above.

Variables were derived from automated measures using Ethovision tracking and were assessed with respect to whole maze or defined maze compartments.

#### 2.6.3 Statistics

Statistical analyses were performed using IBM SPSS, Version 25 (SPSS, Inc., Chicago, IL, USA) beginning with descriptive statistics that included Levine’s *F*-test for equality of variance. Next, comparisons of behavioral data across groups and across testing times were evaluated using analyses of variance (ANOVAs) with repeated measures designs to identify significant main effects of Testing Age (testing repetition), of Sex (comparisons of WT males and females, 3, 5 and 9 months data only) or Genotype (within sex comparisons of WT and *Pink1^–/–^* groups, 3, 5 and 9 months data for males; 3, 5, 7, 9 and 12 months data for females) and significant interactions between these variables. For these comparisons, Mauchly’s test for sphericity of the covariance matrix was applied and degrees of freedom were adjusted as needed using the Huynh-Feldt epsilon. Evidence of significant main effects of Sex or Genotype and/or of significant interactions between Sex or Genotype and Testing Age were explored further using paired-samples *T*-tests to identify test trials (ages) where differences across or genotype were significant. Because there were no *a priori* directional hypotheses for sex, two-sided *t*-tests were used to compare data from WT females to that of WT males. Hypotheses for increased measures of anxiety in *Pink1^–/–^* rats were tested using one-sided *t*-tests that compared data from *Pink1^–/–^* to WT cohorts. Effect sizes were also assessed by calculating Cohen’s D.

## 3 Results

### 3.1 Body weights and estrous cycles

The body weights of wildtype (WT) male and female rats were commensurate with age across the duration of the study. Mean values for both groups have been previously reported [Males (Pinizzotto et al., 2022); Females (Desai et al., 2025)] and data for individual rats are included in the data file that has been made available to readers. Analyses of vaginal cytology samples collected over a 2-weeks period prior to the commencement of behavioral testing confirmed the presence of regular 4-days estrous cycling in all WT and *Pink1^–/–^* female rats, and intermittent cytological and visual performed thereafter confirmed that regular cycling was maintained in all rats for the duration of testing. Determinations of estrous cycle stages on testing days also revealed that with few exceptions, the numbers of rats that were in estrous cycle stages associated with high levels of circulating ovarian steroids (estrus, proestrus) were skewed relative to rats tested during stages when circulating hormone levels were low (diestrus, see Table 2). This negatively impacted the statistical power of planned comparisons of the data stratified by estrous cycle stage which were removed from the study.

**TABLE 2:** Numbers of wild type and *Pink1^–/–^* female rats identified as being in stages of the estrous cycle characterized by relatively low (diestrus) or relatively high (estrus, proestrus) levels of circulating ovarian hormones on the day of Elevated Plus Maze testing at 3, 5, 7, 9 and 12 months of age.

Testing age	Wild type		*Pink1^–/–^*	
	Diestrus	Pro/estrus	Diestrus	Pro/estrus
3 months	7	3	9	3
5 months	7	3	6	6
7 months	8	2	3	9
9 months	10	0	11	1
12 months	9	1	7	3

Estrous cycle stages were identified by vaginal cytology at 3 and 5 months of age, and by visual inspection of the vaginal opening at 7–12 months of age.

### 3.2 Heat maps

Automated overhead tracks of rats’ paths in the elevated plus maze were compiled for each of the four groups evaluated during first exposure to the maze at 3 months of age and for testing at 9 months of age which was the oldest time point that male rats were assessed (Figures 1B, C). These group compilations showed clear differences in the relative amounts of time that 3-months-old WT and *Pink1^–/–^* rats of both sexes spent in different regions of the maze as well as differences in how these spatial maps had changed with repeated testing in rats at 9 months old. The heat maps generated from the tracks of WT males (Figure 1B), for example, showed that these rats spent more time exploring open arms during initial testing compared to testing at 9 months of age. The tracks from WT female rats (Figure 1C), on the other hand, showed greater locomotion than WT males overall, but a relative avoidance of open arms at 3 months of age, and increased exploration of the open compartments at 9 months old. The heat maps for *Pink1^–/–^* rats of both sexes showed greater locomotion and greater amounts of time spent in open arms compared to WT controls in testing at 3 months of age; the *Pink1^–/–^* males also spent more time in maze center than any other group (Figure 1B). Finally, while heat maps for *Pink1^–/–^* males were similar at 3 and 9 months, those for the *Pink1^–/–^* females showed reduced locomotion and markedly reduced times in open arms in testing at 9 compared to 3 months of age and compared to heat maps of age- and sex-matched controls (Figure 1C). These and additional group differences have been defined, quantified and quantitatively compared in analyses below, beginning with assessments made across the plus maze as a whole, followed by evaluations in major maze zones (center space, closed arms, open arms), and finally with respect to proximal, medial and distal thirds of the lengths of the closed and open arm compartments.

### 3.3 Whole maze measures

**FIGURE 2:**
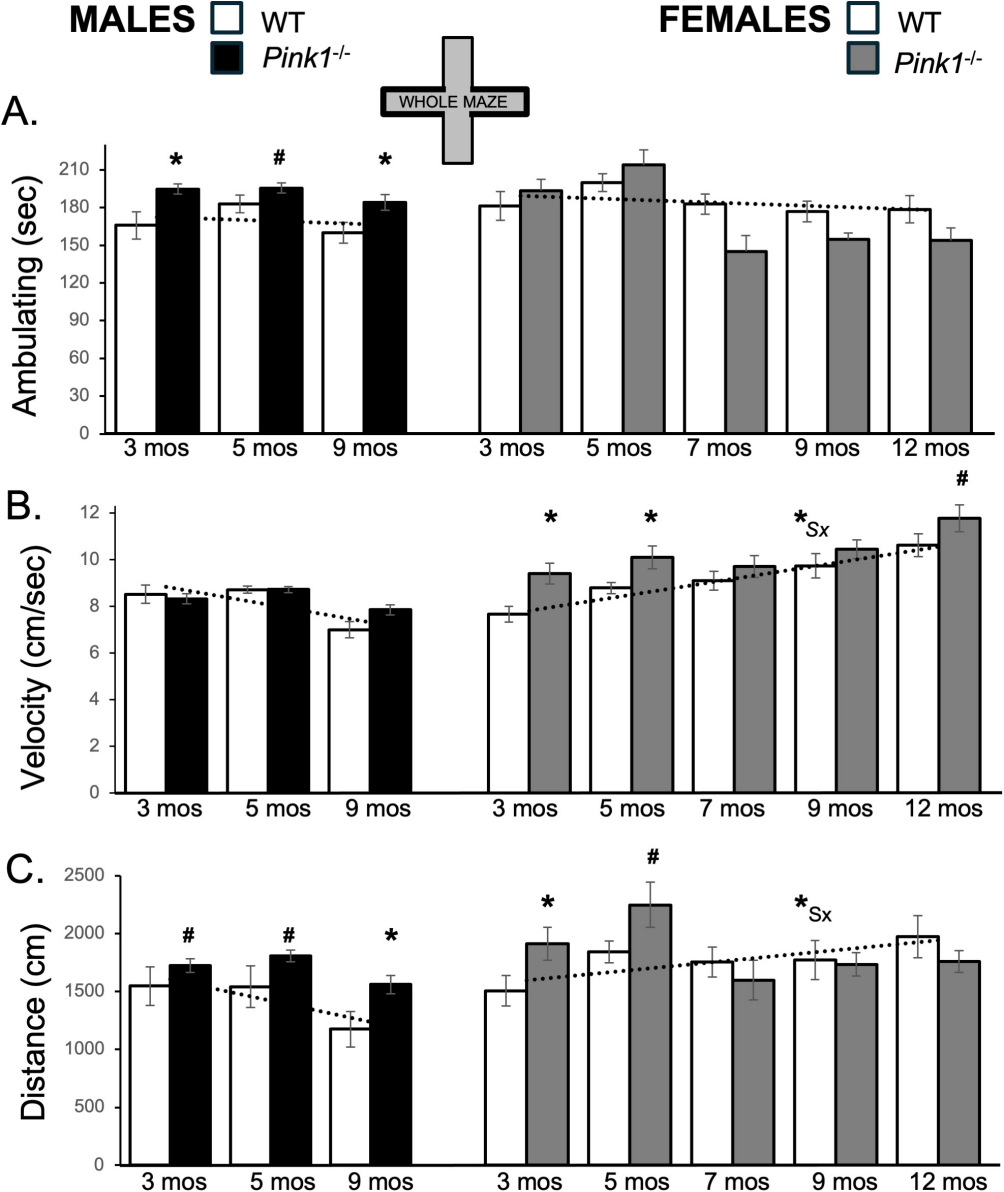
Bar graphs showing average cumulative amounts of time in seconds (sec) that rats in each of the four experimental groups spent ambulating **(A)** in the whole of the elevated plus maze (gray zones, inset figure). Average velocity of ambulation [in centimeters/second (cm/sec) **B**] and average linear distances traveled in the maze [in centimeters (cm), **C**] over the 5-minute trials are also shown. Data from wild type (WT, white bars) and *Pink1^–/–^* (black bars) males, tested at 3, 5 and 9 months (mos) of age are shown in the left column; data from WT (white bars) and *Pink1^–/–^*, gray bars) females, tested at 3, 5, 7, 9 and 12 months of age are shown in the right column. Error bars are standard errors of the mean. For ease of comparison, fitted linear trend lines calculated for the WT groups are shown (dashed lines). Asterisks mark significant differences (*p* < 0.05) within sex between WT and *Pink1^–/–^* rats, hashtags mark near-significant differences (0.05 > *p* < 0.09) within sex between WT and *Pink1^–/–^* rats and asterisks and hashtags superscripted ahead of “Sx” identify data points that are significantly or near significantly different among WT male and female rats.

During initial testing, 3-months-old WT male rats spent roughly 165 s of trial time ambulating (Figure 2A; white bars, left hand column). During ambulation, WT males traveled at an average speed of about 8.5 cm/s and covered total distances of approximately 1500 cm (Figures 2B, C white bars, left hand column). During subsequent re-testing, the average times that the control males spent ambulating remained relatively stable (∼160–180 s; Figure 2A). However, the average speeds of ambulation and the total distances traveled decreased incrementally (Figures 2B, C). Thus, during the last testing session at 9 months of age, WT male rats ambulated at speeds of around 7 cm/s and covered only about 1200 cm of distance (Figures 2B, C). In contrast, WT type female rats ambulated for ∼180–185 s across all trials (Figure 2A; white bars, right hand column). During initial testing, the average speeds of ambulation for WT females (∼7.6 cm/s, Figure 2B) and the average total distance traveled (about 1500 cm, Figure 2C) were similar to those of WT males. However, across repeated testing, both measures progressively increased in WT females, reaching a peak average speed of more than 10 cm/s (Figure 2B) and covering an average total distance of more than 2000 cm in final testing at 12 months of age (Figure 2C). These different trajectories resulted in sex differences in velocity and distances traveled that became larger with repeated testing. This was supported in a series of repeated measures ANOVAs. In addition to identifying significant main effects of testing age/testing repetition on behavioral measures (“Testing Age”) for velocity [F_(2,32)_ = 3.94, *p* = 0.03; η^2^ = 0.20] and distance traveled [F_(2,32)_ = 4.65, *p* = 0.017; η^2^ = 0.23], these comparisons also identified significant interactions between Sex and Testing Age for average velocity [F_(2,32)_ = 37.02, *p* < 0.001; η^2^ = 0.70] and for average total distance traveled [F_(2,32)_ = 7.68, *p* = 0.002; η^2^ = 0.32]. Follow-up pairwise comparisons of values from WT females to males (paired-samples *T*-tests) further showed that sex differences reached significance for velocity and distance traveled in testing at 9 months of age [Velocity: t(7) = −3.54, *p* = 0.010, *d* = −1.25; Distance traveled: t(7) = −2.58, *p* = 0.045, *d* = 0.91, Figures 2B, C].

Whole maze behavioral measures in male *Pink1^–/–^* rats were fairly stable across repeated testing. Thus, average times spent ambulating ranged from about 180 to 220 s (Figure 2A; black bars, left hand column). Male *Pink1^–/–^* rats also maintained average speeds of ambulation of between 7.8 and 9.0 cm/s and covered average total maze distances of between about 1560 and 2050 cm (Figures 2B, C; black bars). The velocities of *Pink1^–/–^* males were thus similar to those of WT males. However, on average, *Pink1^–/–^* males spent more time ambulating and covered more total maze distance than WT male controls. These observations were supported statistically. First, repeated measures ANOVAs identified significant main effects of Testing Age for all behaviors [F_(1.632–2,34.27–4232)_ = 3.91–22.39, *p* = 0.001–0.28; η^2^ = 0.0.15–0.52], and significant to near significant main effects of Genotype for time spent ambulating [F_(1,21)_ = 7.30, *p* = 0.013; η^2^ = 0.26] and total distance traveled [F_(1,21)_ = 4.25, *p* = 0.052; η^2^ = 0.17]. Allowed *post hoc* comparisons (paired-samples *T*-tests) further showed that differences across genotype were significant to near significant for ambulation and distance traveled at all ages [t(6-7) = −1.42 to −5.44, *p* = 0.001–0.099, *d* = −0.50 to −2.06, Figures 2A, C].

Whole maze behavioral measures in female *Pink1*^–/^rats (Figure 2; gray bars, right hand columns) showed several abrupt changes over time. First, average velocities of ambulation were 9.3 and 10.0 cm/s in testing at 3 and 5 months of age, respectively. However, average speed dropped to around 9.6 cm/s in testing at 7 months of age before rising incrementally in testing at 9 and 12 months of age to reach maximum average velocities of approximately 11.6 cm/s (Figure 2B; gray bars). The average times that female *Pink1^–/–^* rats spent ambulating were also about 196 and 217 s during the first two trials but were noticeably less (∼147–157 s) for the final three testing sessions (Figure 2A; gray bars). Total distances traveled followed a similar pattern, with initial values of 1947 and 2286 cm in testing at 3 and 5 months of age dropping to distances of between about 1626 and 1765 cm in testing at 7, 9 and 12 months of age (Figure 2C; gray bars). Thus, during initial testing, female *Pink1^–/–^* rats spent more time ambulating, ambulated at higher speeds and covered more ground than WT female controls. However, in testing from 7 months on, *Pink1^–/–^* females spent similar amounts of time ambulating, traveled at similar speeds and covered slightly less ground overall than WT females. Other than main effects of Testing Age for all measures [F_(4,76)_ = 4.33–20.02, *p* = 0.001–0.003; η^2^ = 0.19–0.51], however, repeated measures ANOVAs only identified significant interactions between Genotype and Testing Age for average distance traveled [F_(4,76)_ = 2.85, *p* = 0.03; η^2^ = 0.13] and near significant main effects of Genotype for average velocity of ambulation [F_(1,19)_ = 4.04, *p* = 0.059; η^2^ = 0.18]. *Post hoc* comparisons similarly identified significant to near significant group/genotype differences for average velocities in testing at 3, 5 and 12 months of age [t(9) = −1.50 to −3.32, *p* = 0.004–0.084, *d* = −0.48 to −1.05, Figure 2B] and significant to near-significant group differences in distance traveled in testing at 3 and 5 months of age [t(9) = −1.56 to −2.58, *p* = 0.015–0.077, *d* = −0.49 to −0.82, Figure 2C].

### 3.4 Total time spent per maze compartments

#### 3.4.1 Central platform

**FIGURE 3:**
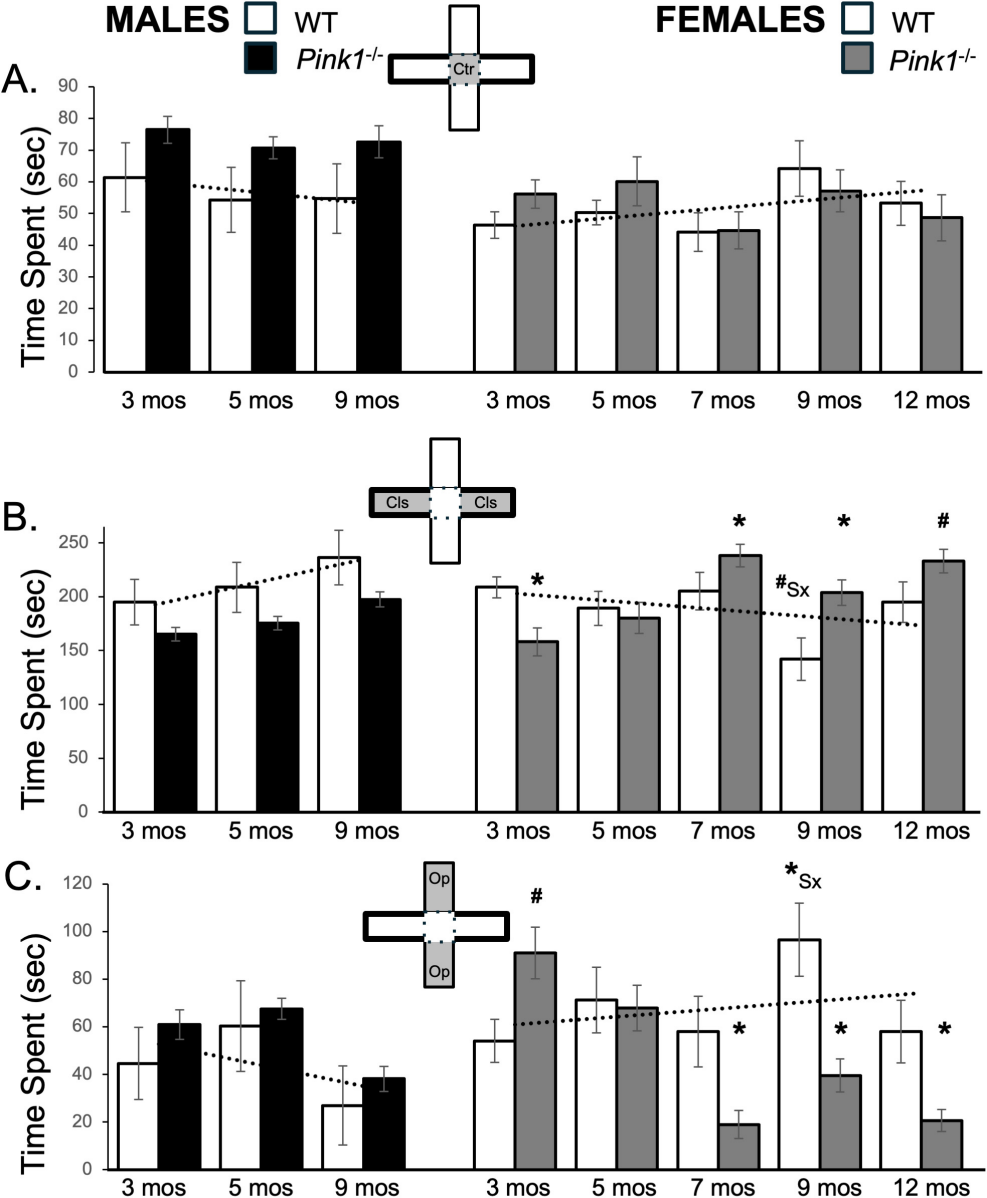
Bar graphs showing average amount cumulative amounts of time in seconds (s) that rats in each of the four experimental groups spent in the maze center (**A**, gray zone, inset figure) in closed arms of the maze (**B**, gray zones, inset figure) or in open arms of the maze (**C**, gray zones, inset figure). For ease of visual comparison across compartments, the data are plotted on the same scale. Data from wild type (WT, white bars) and *Pink1^–/–^* (black bars) males, tested at 3, 5 and 9 months (mos) of age are shown in the left column; data from WT (white bars) and *Pink1^–/–^*, gray bars) females, tested at 3, 5, 7, 9 and 12 months of age are shown in the right column. Error bars are standard errors of the mean. For ease of comparison, fitted linear trendlines calculated for the WT groups are shown (dashed lines). Asterisks mark significant differences (*p* < 0.05) within sex between WT and *Pink1^–/–^* groups and asterisks superscripted ahead of “Sx” identify data points that are significantly different among WT male and female rats.

From trial to trial, most rats spent about 45–55 s in the central platform of the maze. Only the *Pink1^–/–^* male rats consistently spent noticeably more time in this compartment (70–76 s, Figure 3A; black bars, Figure 1A). These observations were supported in a series of repeated measures ANOVAs that only identified significant main effects of Genotype on center maze time, and only for the male rats [F_(1,21)_ = 4.89, *p* = 0.038; η^2^ = 0.19]. Follow-up pairwise comparisons however, found no significant group/genotype differences in average times spent WT and *Pink1^–/–^* male rats spent within the maze center.

#### 3.4.2 Closed arms

During first testing experiences, male and female WT rats spent about 200 s in closed arms of the maze (Figure 3B; white bars). Although variable, as testing was repeated WT males spent progressively more time and WT females progressively less time in closed arm zones (Figure 3B; white bars). This produced sex differences in average closed arm occupancies that became larger over time. A repeated measures ANOVA found no main effects of Testing Age but did confirm the progressive differences in closed arm times in WT males and females in findings of significant interactions between Sex and Testing across these groups [F_(2,32)_ = 6.72, *p* = 0.004; η^2^ = 0.30]. Follow up pairwise comparisons further showed that sex differences in this measure were near-significant for testing at 9 months of age [t(7) = 2.20, *p* = 0.064, *d* = 0.78, Figure 3B]. In contrast, 3-months-old male and female *Pink1^–/–^* rats spent an average of about 150 s in the closed arms, i.e., almost 1 min less than the average times spent by age- and sex- matched WT controls (Figure 3B; black, gray bars). As repeated testing continued, the average amount of time that *Pink1^–/–^* males spent in closed arms incrementally increased but remained below the corresponding times for WT males (Figure 3B; black bars). A repeated measures ANOVA, however, found significant main effects of Testing Age [F_(2,42)_ = 8.51, *p* < 0.001; η^2^ = 0.29], but no significant or near significant main effects of Genotype and no significant or near significant interactions between Genotype and Testing Age for average total time spent in closed arms. Finally, unlike WT females–but similar to *Pink1^–/–^* males, female *Pink1^–/–^* rats showed gradual increases in times spent in the closed arms during testing at 3–5 months of age (∼158–180 s, Figure 3B; gray bars), and larger increases in times spent in these compartments in testing from 7 months of age on, when the average amount of time that *Pink1^–/–^* females spent in the closed arms was between roughly 200 and 240 s– considerably than corresponding times spent by WT female rats (Figure 3B; gray bars). A repeated measures ANOVA confirmed that in addition to significant main effects of Testing Age [F_(4,76)_ = 6.96, *p* < 0.001; η^2^ = 0.27], there were also significant interactions between Genotype and Behavior among the two female groups [F_(4,76)_ = 7.67, *p* < 0.001; η^2^ = 0.29]. Follow up pairwise comparisons further showed that the average times that female *Pink1^–/–^* rats spent in closed maze arms were significantly shorter than controls in testing at 3 months of age [t(9) = 2.82, *p* = 0.010, *d* = 0.89, Figure 3B], and significantly to near significantly longer than controls in testing at 5, 7 and 9 months of age [t(9) = −1.67 to −3.30, *p* = 0.005–0.065, *d* = −0.52 to −1.04, Figure 3B].

#### 3.4.3 Open arms

Wild type male and female rats initially spent about 44 and 54 s, respectively, in open arms of the plus maze (Figure 3C; white bars). Both WT groups also explored this compartment slightly more (∼60 and 71 s) in testing at 5 months of age. Thereafter, WT males reduced average times spent in open arms (27 s), while WT females spent similar to more time (∼ 57–97 s) in these spaces (Figure 3C, white bars). These differences were reflected in findings from a repeated measures ANOVA; although there were no significant main effects of Testing Age, significant interactions between Sex and Behavior were found for average open arm times among the two WT groups [F_(2,32)_ = 5.64, *p* = 0.008; η^2^ = 0.26]. Follow-up comparisons further confirmed that sex differences in average open arm times reached significance in testing at 9 months of age [t(7) = −2.67, *p* = 0.032, *d* = −0.94, Figure 3C].

Male *Pink1^–/–^* rats generally spent similar average amounts of time in open arms as the male WT controls (3 months, ∼ 61 s; 5 months, ∼67 s; 9 months, ∼38 s, Figure 3C; black bars). These similarities were confirmed in a repeated measures ANOVA that identified significant main effects of Testing Age [F_(2,42)_ = 14.88, *p* < 0.001; η^2^ = 0.42], but no significant or near significant main effects of Genotype and no significant or near significant interactions between Genotype and Testing Age for this measure. In contrast, female *Pink1^–/–^* rats initially spent longer in open arms than WT females (∼85 vs. 54 s, Figure 3C; gray bars). However, at 5 months of age, the *Pink1^–/–^* females reduced times spent in this compartment to values that were similar to WT females (∼60 s) and from 7 months on, the *Pink1^–/–^* females reduced average open arm times further to values that were lower than controls (∼18 to −37 s). In addition to main effects of Testing Age [F_(4,76)_ = 7.70, *p* < 0.001; η^2^ = 0.29], a repeated measures ANOVA confirmed that there were significant interactions between Genotype and Testing Age [F_(1,19)_ = 20.51, *p* < 0.001; η^2^ = 0.52] and a near significant main effect of Genotype [F_(1,19)_ = 3.81, *p* = 0.066; η^2^ = 0.16] on average open arm times among the female groups. Pairwise comparisons also showed that female *Pink1^–/–^* rats spent significantly more time in open arms than WT females in testing at 3 months [t(9) = −2.18, *p* = 0.028, *d* = −0.69, Figure 3C] and significantly less time in open arms than female controls in testing at 7, 9 and 12 months of age [t(9) = 2.52–3.94, *p* = 0.002–0.017, *d* = 0.73–1.14, Figure 3C].

### 3.5 Numbers of maze compartment entries

#### 3.5.1 Center platform entries

**FIGURE 4:**
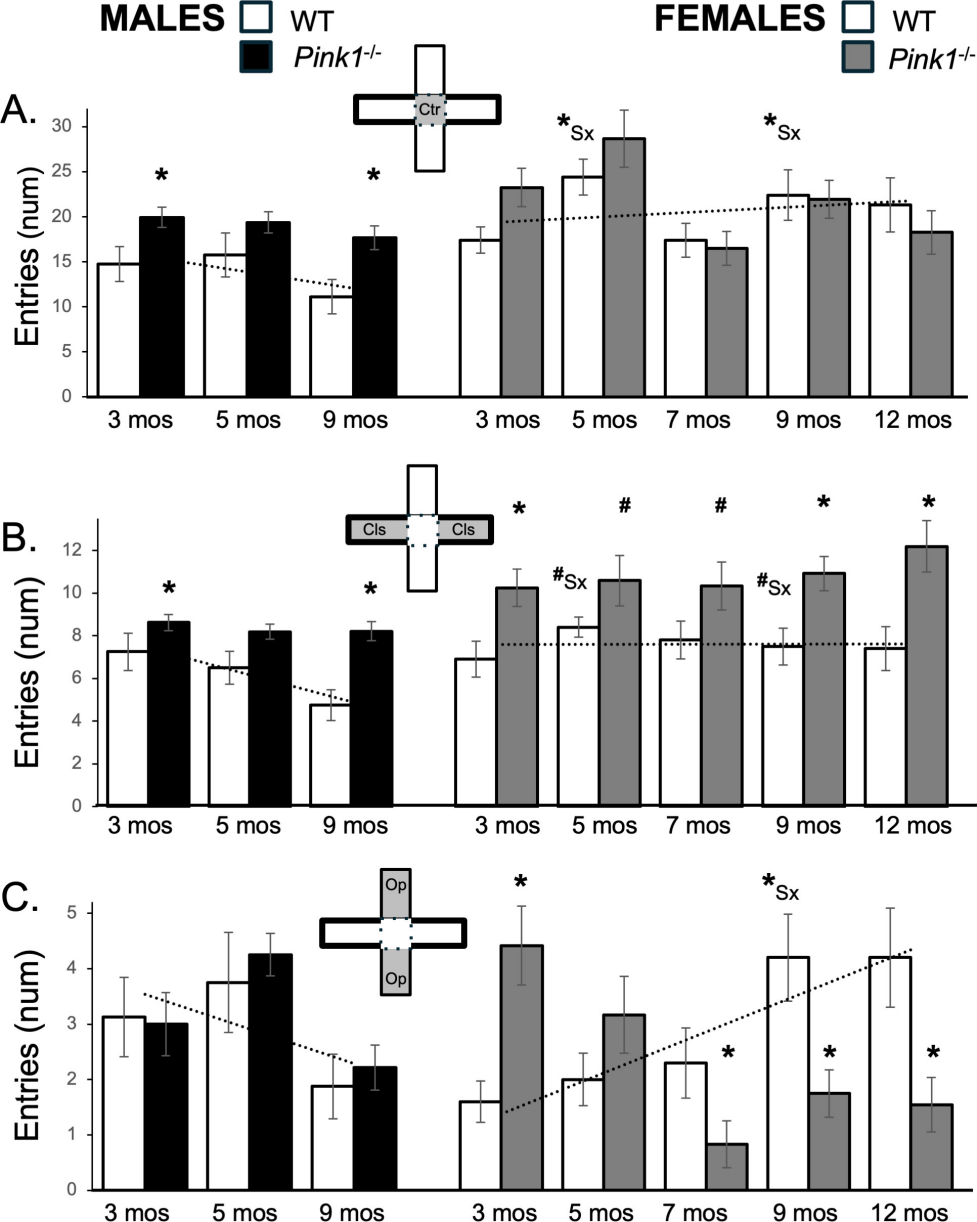
Bar graphs showing average numbers (num) of entries that rats in each of the four experimental groups made into the maze center (**A**, gray zone, inset figure), into closed arms of the maze (**B**, gray zones, inset figure) or into open arms of the maze (**C**, gray zones, inset figure). Data from wild type (WT, white bars) and *Pink1^–/–^* (black bars) males, tested at 3, 5 and 9 months (mos) of age are shown in the left column; data from WT (white bars) and *Pink1^–/–^*, gray bars) females, tested at 3, 5, 7, 9 and 12 months of age are shown in the right column. Error bars are standard errors of the mean. For ease of comparison, fitted linear trendlines calculated for the WT groups are shown (dashed lines). Asterisks mark significant differences (*p* < 0.05) within sex between WT and *Pink1^–/–^* groups and asterisks superscripted ahead of “Sx” identify data points that are significantly different among WT male and female rats.

On average, 3-months-old male and female WT rats crossed into the center arena platform an average of ∼15 and 18 times, respectively (Figure 4A; white bars). However, over subsequent repeated testing the average numbers of center platform entries decreased in WT males to lows of ∼11 entries and increased in WT females to highs of ∼22 entries or more (Figure 4A; white bars). This produced female over male differences in center space entries that increased over time. A repeated measures ANOVA confirmed that in addition to main effects of Testing Age [F_(1.59,25.49)_ = 2.89, *p* = 0.084; η^2^ = 0.15] and interactions between Sex and Testing Age that approached significance [F_(1.59,25.49)_ = 3.09, *p* = 0.073; η^2^ = 0.16], there were also significant main effects of Sex [F_(1,16)_ = 10.92, *p* = 0.004; η^2^ = 0.41] on center space entries for the WT groups. *Post hoc* paired comparisons further showed that sex difference in the number of center space entries reached significance in testing at 5 and 9 months of age [t(7) = −2.54 to −3.11, *p* = 0.009–0.019, *d* = −0.90 to −1.10, Figure 4A]. Male *Pink1^–/–^* rats, on the other hand, consistently entered the central platform an average of ∼17–20 times, i.e., ∼ 5–6 more times than the entries of WT males (Figure 4A; black bars). These differences were confirmed in a repeated measures ANOVA that identified significant main effects of Testing Age [F_(2,42)_ = 3.74, *p* = 0.032; η^2^ = 0.15] and significant main effects of Genotype on this measure [F_(1,21)_ = 10.36, *p* = 0.004; η^2^ = 0.33]. Follow-up pairwise comparisons further showed that group differences were significant in testing at 3 and 9 months of age [t(7) = −2.35 to −2.80, *p* = 0.013–0.026, *d* = −0.83 to −0.99, Figure 4A]. At 3 and 5 months old, female *Pink1^–/–^* rats also made roughly 4–5 more average entries into the center space than WT females (∼23–29 vs. ∼18–25, respectively, Figure 4A; gray bars). However, from 7 months on, the numbers of times female *Pink1^–/–^* rats entered the central platform dropped to between 16 and 22 entries, which were marginally lower than entries made by sex- and age- matched controls (Figure 4A; gray bars). However, although significant main effects of Testing Age were found [F_(2.91,52.47)_ = 5.90, *p* = 0.002; η^2^ = 0.25], a repeated measures ANOVA found no significant main effects of Genotype and no significant interactions between Genotype and Behavior on average measures of center platform entries for the two female groups.

#### 3.5.2 Closed arm entries

Wild type male and female rats made an average of about 7 entries into closed arms during initial testing (Figure 4B; white bars) Thereafter, WT male rats decreased the numbers of closed arms entries to ∼ 5 while WT females continued to make similar to slightly more closed arm entries (∼7–8.0, Figure 4B; white bars) across remaining sessions. This produced female over male sex differences in closed arm entries that were most evident for later testing timepoints. These observations were supported in a repeated measures ANOVA that identified significant main effects of Testing Age [F_(2,32)_ = 3.89, *p* = 0.031; η^2^ = 0.20], and significant interactions between Testing Age and Sex [F_(2,32)_ = 5.35, *p* = 0.010; η^2^ = 0.25] and in follow-up pairwise comparisons showing that sex differences in closed arm entries approached significance in testing at 5 and 9 months of age [t(7) = −1.92 to −2.11, *p* = 0.073–0.097, *d* = −0.66 to −0.68, Figure 4B].

The closed arm entries made by male and female *Pink1^–/–^* rats (∼8–12) were more numerous than those made by age- and sex-matched controls (Figure 4B; black, gray bars). Differences among the male groups were confirmed in a repeated measures ANOVA that identified significant main effects of Testing Age [F_(1.64,36.1)_ = 6.33, *p* = 0.007; η^2^ = 0.22] and Genotype [F_(1,22)_ = 12.27, *p* = 0.002; η^2^ = 0.36] and a significant interaction between these two [F_(.64,36.1)_ = 3.58, *p* = 0.046; η^2^ = 0.14], and were further supported in follow up pairwise comparisons that showed that group differences between *Pink1^–/–^* and WT control males were significant for testing at 3 and 9 months of age [t(7) = −1.90 to −4.97, *p* = 0.017–0.050, *d* = −0.67 to −1.76, Figure 4B]. Statistical support for differences in the females included repeated measures ANOVA findings of significant main effects of Genotype [F_(1,20)_ = 10.29, *p* = 0.004; η^2^ = 0.34] and follow up pairwise comparisons showing that group differences between *Pink1^–/–^* and WT control females were significant for testing at 3, 9 and 12 months of age [t(9) = −2.50 to −3.45, *p* = 0.017−0.004, *d* = −0.79 to −1.09, Figure 4B] and near significant for testing at 5 and 7 months of age [t(9) = −1.45 to −1.73, *p* = 0.058–0.091, *d* = −0.46 to −0.55, Figure 4B].

#### 3.5.3 Open arm entries

During testing at 3 and 5 months, WT males entered open arms more often than WT females (∼1.6 vs. 3 times, respectively, Figure 4C; white bars). However, over time entries decreased in WT males and increased in WT females, thus producing sex differences in average numbers of open arm entries that grew with repeated testing. These findings were supported in a repeated measures ANOVA that found significant interactions between Sex and Testing Age [F_(2,32)_ = 12.39, *p* < 0.001; η^2^ = 0.44] and in follow up pairwise comparisons showing that sex differences in open arm entries were significant in testing at 9 months of age [t(7) = −2.46, *p* = 0.044, *d* = −0.87, Figure 4C].

The numbers of open arm entries made by *Pink1^–/–^* males (Figure 4C; black bars) were similar to those of WT males at all ages. This was confirmed in a repeated measures ANOVA that identified significant main effects of Testing Age [F_(2,40)_ = 9.38, *p* < 0.001; η^2^ = 0.32], but no significant or near significant main effects of Genotype and no significant or near significant interactions between Genotype and Testing Age. In contrast, the average numbers of open arm entries made by female *Pink1^–/–^* rats (Figure 4C; gray bars) were highest during testing at 3 months of age (∼5), dropped slightly in testing at 5 months (∼3) and dropped further to average values of between roughly 0.8 and 1.5 open arm entries in testing from 7 to 12 months of age. Thus, the numbers of open arm entries made by the *Pink1^–/–^* females went from values that were greater than WT to ones that were lower. These observations were supported in a repeated measures ANOVA that identified significant main effects of Testing Age [F_(4,76)_ = 3.62, *p* = 0.009; η^2^ = 0.16] and significant interactions between Testing Age and Genotype [F_(4,76)_ = 15.22, *p* < 0.001; η^2^ = 0.45] for these data. Follow up pairwise comparisons further showed that open arm entries were significantly greater in *Pink1^–/–^* females compared to WT females at 3 months of age [t(9) = −4.11, *p* = 0.001, *d* = −1.30, Figure 4C] and significantly lower than female controls in testing at 7, 9 and 12 months of age [t(9) = 1.89–3.63, *p* = 0.003–0.045, *d* = 0.60–1.15, Figure 4C].

### 3.6 Center platform measures

#### 3.6.1 Ambulating

**FIGURE 5:**
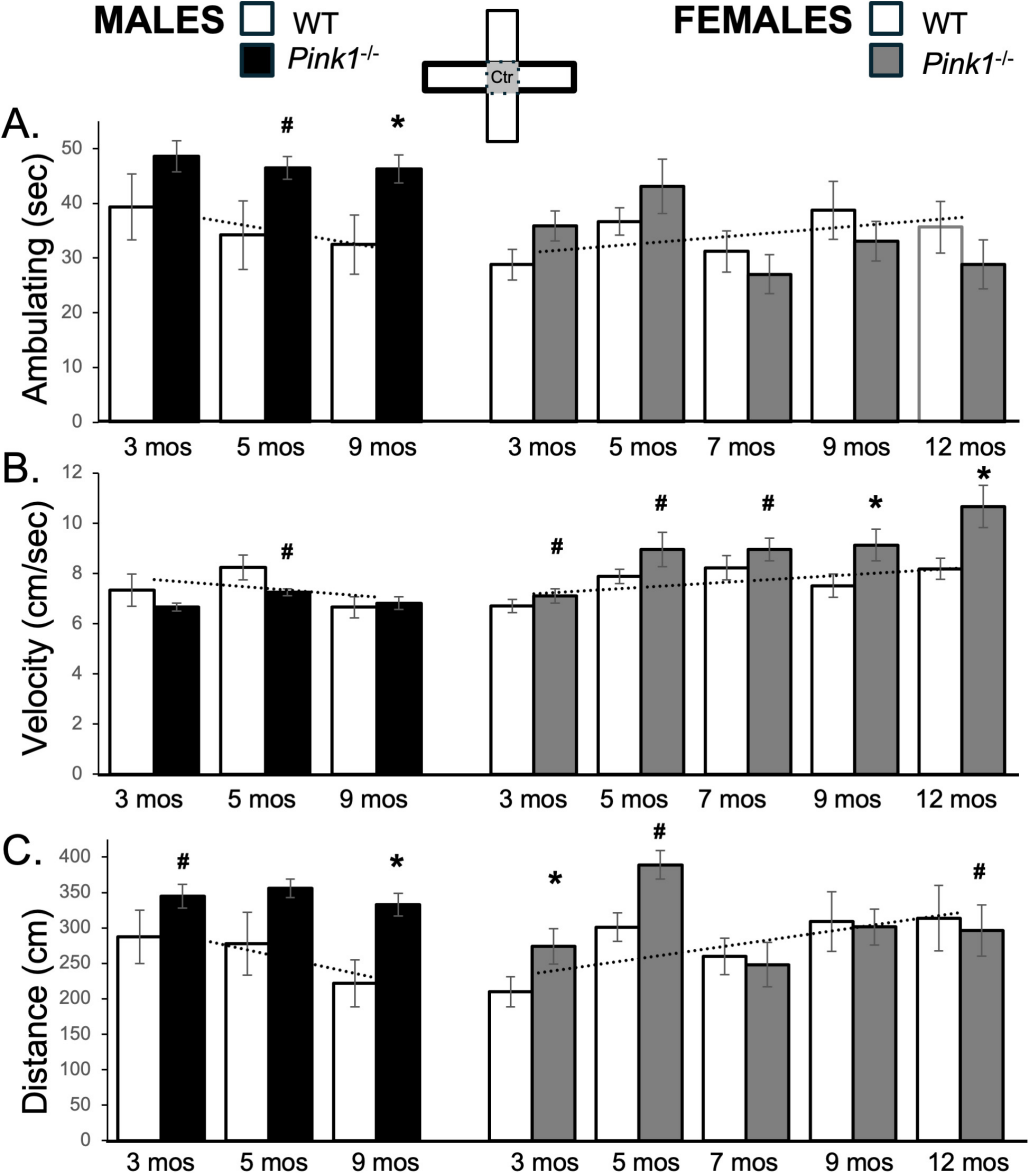
Bar graphs showing average cumulative amounts of time in seconds (s) that rats in each of the four experimental groups spent ambulating **(A)** to time in the center platform of the elevated plus maze (gray zone, inset figure). Average velocity of ambulation [in centimeters/second (cm/s) **B**] and average linear distances traveled in the maze [in centimeters (cm), **C**] over the 5-min trials are also shown. Data from wild type (WT, white bars) and *Pink1^–/–^* (black bars) males, tested at 3, 5 and 9 months (mos) of age are shown in the left column; data from WT (white bars) and *Pink1^–/–^*, gray bars) females, tested at 3, 5, 7, 9 and 12 months of age are shown in the right column. Error bars are standard errors of the mean. For ease of comparison, fitted linear trendlines calculated for the WT groups are shown (dashed lines). Asterisks mark significant differences (*p* < 0.05) within sex between WT and *Pink1^–/–^* rats, hashtags mark near-significant differences (0.05 > *p* < 0.09) within sex between WT and *Pink1^–/–^* rats and asterisks and hashtags superscripted ahead of “Sx” identify data points that are significantly or near significantly different among WT male and female rats.

The average amounts of time that WT rats spent ambulating were initially about 40 s for males and less than 30 s for females (Figure 5A; white bars). However, across repeated testing, average ambulation decreased in WT males and increased in WT females. Thus, by testing at 9 months of age, WT rats of both sexes ambulated in the central space for ∼35 s (Figure 5A; white bars). A repeated measures ANOVA that compared these data found no significant or near significant main effects of Testing Age or Sex, but did find interactions between Sex and Behavior that approached significance [F_(2,32)_ = 2.86, *p* = 0.072; η^2^ = 0.15]. However, follow-up comparisons found no times when sex differences in this measure were significant or near significant across the two WT groups.

Male *Pink1^–/–^* rats consistently spent about 10–15 s longer ambulating in the maze center than sex-matched controls (∼41–49 s vs. ∼32–39 s, respectively, Figure 5A; black bars). These differences were confirmed in a repeated measures ANOVA that identified significant main effects of Genotype [F_(1,21)_ = 8.08, *p* = 0.010; η^2^ = 0.28] and in follow up pairwise comparisons that identified significant differences in the times that *Pink1^–/–^* vs. WT males ambulated in the maze center in testing at 9 months of age [t(7) = −4.93, *p* < 0.001, *d* = −1.74, Figure 5A] and near significant differences in the times that *Pink1^–/–^* vs. WT males ambulated in the maze center in testing at 5 months of age [t(7) = −1.51, *p* < 0.088, *d* = −1.26, Figure 5B]. At 3 and 5 months old, *Pink1^–/–^* female rats also spent more time ambulating in the maze center than WT females (∼36–44 s vs. ∼29–36 s, respectively, Figure 5A; gray bars). However, at 7 months of age and older, center space ambulation decreased in the *Pink1^–/–^* females to times that were about 4–5 s less than those of WT females (∼27–32 s, Figure 5A; gray bars). Thus, while a repeated measures ANOVA identified significant main effects of Testing Age [F_(4,76)_ = 3.87, *p* = 0.006; η^2^ = 0.17] and significant interactions between Genotype and Testing Age for this measure [F_(4,76)_ = 2.83, *p* = 0.031; η^2^ = 0.13], follow up pairwise comparisons found no significant differences in center maze ambulation times for any testing session.

#### 3.6.2 Velocity and distance traveled

The average velocity of ambulation in the maze center for WT male rats was initially slightly faster than that of WT females (∼7.3 cm/s vs. 6.7 cm/s, Figure 5B; white bars). However, across repeated testing, average speeds slowed in WT males and increased in WT females, thus keeping the absolute differences in velocities between these two groups small. This was reflected in a repeated measures ANOVA that identified significant main effects of Testing Age [F_(2,32)_ = 7.52, *p* = 0.002; η^2^ = 0.32] and significant interactions between Behavior and Sex [F_(2,32)_ = 2.83, *p* = 0.044; η^2^ = 0.18] but no significant main effects of Sex. Follow up pairwise comparisons also found no instances where sex differences in this measure were significant or near significant.

The average speeds of male *Pink1^–/–^* rats were mostly similar to those of age-matched WT males (Figure 5B; black bars); the only exception was for testing at 5 months of age when the *Pink1^–/–^* males slowed to speeds that were lower than those of age- and sex-matched controls (∼7.6 cm/s vs. ∼8.2 cm/s). These observations were supported in a repeated measures ANOVA that identified significant main effects of Testing Age [F_(2,42)_ = 9.72, *p* < 0.001; η^2^ = 0.32] and near significant interactions between Genotype and Testing Age [F_(2,42)_ = 2.70, *p* = 0.079; η^2^ = 0.114]. Follow up pairwise comparisons that that differences in average velocity at the 5 months timepoint approached significance [t(7) = 1.81, *p* = 0.057, *d* = 0.64, Figure 5C]. Average ambulation speeds in female *Pink1^–/–^* rats, on the other hand, were greater than those of WT females, particularly in testing at 9 and 12 months of age (Figure 5B; gray bars). A repeated measures ANOVA confirmed that in addition to significant main effects of Testing Age [F_(4,76)_ = 8.68, *p* < 0.001; η^2^ = 0.31, there were also significant main effects of Genotype for center maze velocity measures in the female groups [F_(1,19)_ = 6.32, *p* = 0.021; η^2^ = 0.25]. Follow up pairwise comparisons further confirmed that group differences in this measure were significant in testing at 9 and 12 months of age [t(9) = −3.19 to −3.24, *p* = 0.005–0.006, *d* = −1.007 to −1.0251, Figure 5B] and were near significant in testing at 3, 5 and 7 months of age [t(9) = −1.54 to −1.58, *p* = 0.074–0.079, *d* = −0.49 to −0.50, Figure 5B].

In all groups, trends in average total distances traveled tracked closely with ambulation times. Thus, the distances traveled by WT males were initially higher than those of WT females (∼288 cm vs. 210 cm) and decreased (∼287–220 cm) over time while distances traveled by WT females increased (∼300–308 cm) with repeated testing (Figure 5C; white bars). These patterns brought distance values in WT males and females closer together over time. These observations were supported in a repeated measures ANOVA that identified significant main effects of Testing Age [F_(2,42)_ = 9.72, *p* < 0.001; η^2^ = 0.32] and near significant interactions between Genotype and Testing Age [F_(2,42)_ = 2.70, *p* = 0.079; η^2^ = 0.11]. However, follow up comparisons found no sex differences in this measure at any age that were significant or near significant. In contrast, the average distances traveled by male *Pink1^–/–^* rats were consistently some 60–100 cm longer than those of WT males (Figure 5C; black bars). In addition to significant main effects of Testing Age [F_(2,42)_ = 3.43, *p* = 0.042; η^2^ = 0.14], a repeated measures ANOVA comparing these data also identified significant main effects of Genotype for this measure [F_(1,21)_ = 481.16, *p* < 0.001; η^2^ = 0.96]. Follow up pairwise comparisons further showed that group/genotype differences in average distances travels among *Pink1^–/–^* and WT male rats were significant in testing at 9 months of age [t(7) = −4.32, *p* = 0.002, *d* = −1.53, Figure 5C] and near significant in testing at 3 months of age [t(7) = −1.60, *p* = 0.077, *d* = −0.57, Figure 5C]. In female *Pink1^–/–^* rats however, average distances that were ∼70–100 cm greater than WT females at 3 and 5 months of age, dropped to distances that were similar to those of WT female rats in testing at 7, 9 and 12 months of age (Figure 5C; gray bars). Thus, while a repeated measures ANOVA identified near significant main effects of Testing Age [F_(1.70,32.37)_ = 2.96, *p* = 0.073; η^2^ = 0.14] and significant interactions between Testing Age and Genotype [F_(1.70,32.37)_ = 3.55, *p* = 0.047; η^2^ = 0.16], subsequent pairwise comparison showed that differences were only significant for testing at 3 months of age [t(9) = −2.39, *p* = 0.020, *d* = −0.76, Figure 5C] but were near significant for testing at 5 and 12 months of age [t(9) = −1.58, *p* = 0.075, *d* = −0.46 to 0.50, Figure 5C]

### 3.7 Closed arm measures

#### 3.7.1 Ambulation

**FIGURE 6:**
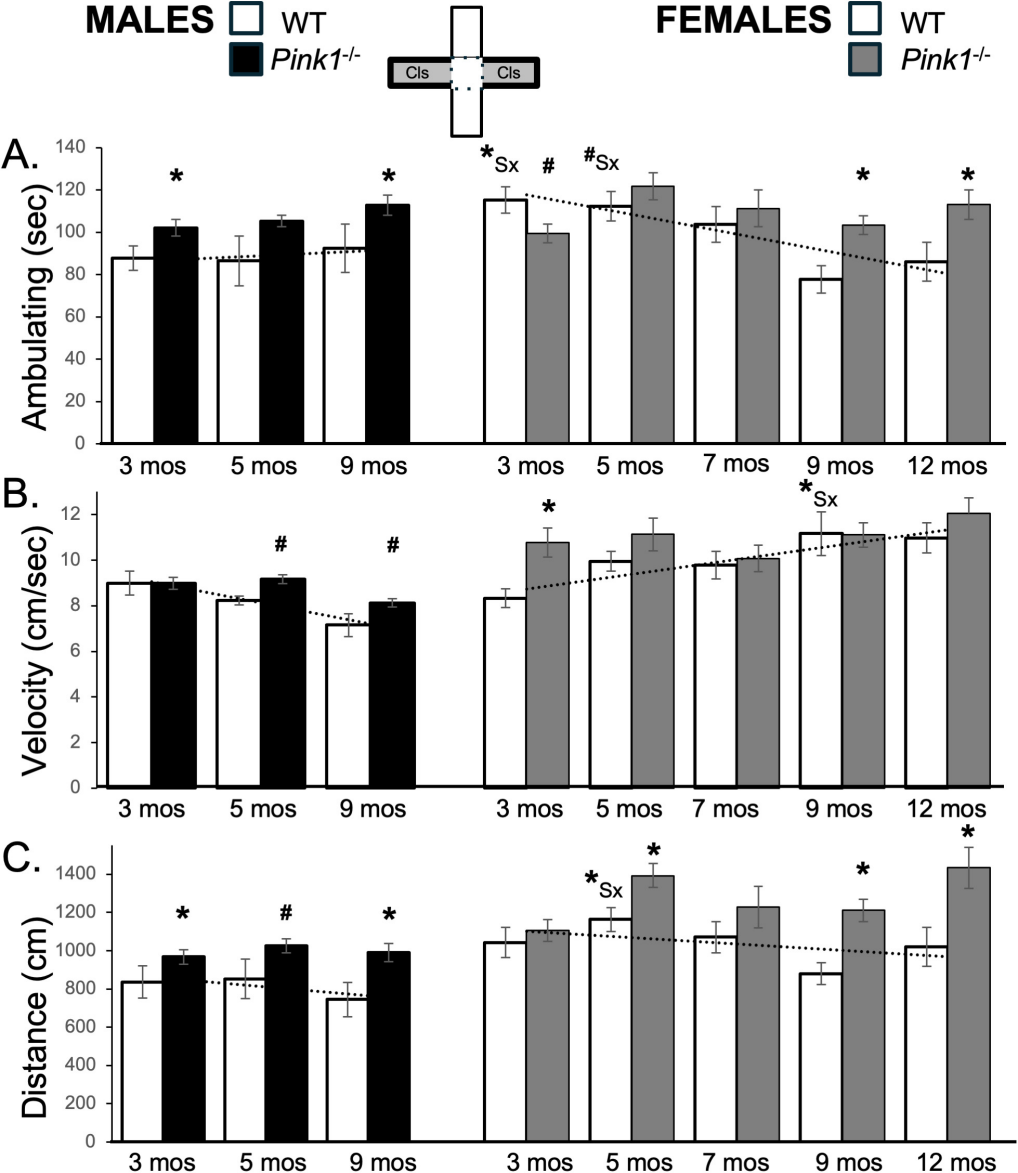
Bar graphs showing average cumulative amounts of time in seconds (s) that rats in each of the four experimental groups spent ambulating **(A)** to time in the closed arms of the elevated plus maze (gray zones, inset figure). Average velocity of ambulation [in centimeters/second (cm/s) **B**] and average linear distances traveled in the maze [in centimeters (cm), **C**] over the 5-min trials are also shown. Data from wild type (WT, white bars) and *Pink1^–/–^* (black bars) males, tested at 3, 5 and 9 months (mos) of age are shown in the left column; data from WT (white bars) and *Pink1^–/–^*, gray bars) females, tested at 3, 5, 7, 9 and 12 months of age are shown in the right column. Error bars are standard errors of the mean. For ease of comparison, fitted linear trendlines calculated for the WT groups are shown (dashed lines). Asterisks mark significant differences (*p* < 0.05) within sex between WT and *Pink1^–/–^* rats, hashtags mark near-significant differences (0.05 > *p* < 0.09) within sex between WT and *Pink1^–/–^* rats and asterisks and hashtags superscripted ahead of “Sx” identify data points that are significantly or near significantly different among WT male and female rats.

Wild type male rats consistently spent between 87 and 92 s ambulating. These times were shorter than those for the WT females (∼115 s) at 3 months of age (Figure 6A; white bars). However, over time ambulation in WT females decreased to durations that were similar to those of WT males (∼ 77–85 s). These observations were supported in repeated measures ANOVAs. In addition to significant main effects of Testing Age for [F_(2,32)_ = 3.47, *p* = 0.043; η^2^ = 0.18], these analyses also identified significant interactions between Sex and Testing Age [F_(2,32)_ = 6.27, *p* = 0.005; η^2^ = 0.28]. Follow-up pairwise comparisons further showed that sex differences in average ambulation times were significant in testing at 3 months [t(7) = −4.70, *p* = 0.002, *d* = −1.66, Figure 6A] and near significant in testing at 5 months of age [t(7) = −2.10, *p* = 0.075, *d* = −0.74, Figure 6A].

At every testing age, male *Pink1^–/–^* rats ambulated ∼10–20 s more than control males in closed arm spaces (Figure 6A; black bars. This was confirmed in a repeated measures ANOVA that identified significant main effects of Genotype on this measure [F_(1,21)_ = 7.23, *p* = 0.014; η^2^ = 0.98] and in follow up pairwise comparisons that showed that differences among WT and *Pink1^–/–^* males were significant for testing at 3 and 9 months of age [t(7) = −2.07 to −2.39, *p* = 0.024–0.039, *d* = −0.48 to −0.73, Figures 6A, B]. For female *Pink1^–/–^* rats, average times spent ambulating were steady and ranged from ∼100 to 113 s (Figure 6A, gray bars). This yielded ambulation times that were initially shorter than those of WT females but became longer as ambulation times in WT group progressively declined (Figure 6A). These differences were confirmed in repeated measures ANOVAs that identified significant main effects of Testing Age [F_(4,76)_ = 4.88, *p* < 0.001; η^2^ = 0.20], significant interactions between Testing Age and Genotype [F_(4,76)_ = 3.50, *p* = 0.011; η^2^ = 0.16 and near significant main effects of Genotype [F_(1,19)_ = 4.09, *p* = 0.058; η^2^ = 0.18]. Follow up pairwise comparisons further showed that group/genotype differences were significant for ambulation in testing at 3 and 9 months of age [t(7) = −2.07 to −2.39, *p* = 0.024–0.039, *d* = −0.73 to 0.85 Figure 6A].

#### 3.7.2 Velocity and distance traveled

The average speeds of ambulation were initially similar in WT males and females (∼8.7 and 8.3 cm/s, respectively) but slowed in WT males and increased in WT females over time (Figure 6B; white bars). A repeated measures ANOVA confirmed that in addition to significant main effects of Testing Age, there were also significant main effects of Sex [F_(1,17)_ = 3.97, *p* = 0.029; η^2^ = 0.27] and significant interactions between Sex and Testing Age [F_(2,32)_ = 11.93, *p* = < 0.001; η^2^ = 0.43] for this measure. Pairwise comparisons further showed that sex differences in average velocities reached significance in testing rats at 9 months old [t(7) = −2.95, *p* = 0.022, *d* = −1.04, Figure 6B]. Finally, the average total distances traveled were initially lower in WT males compared to WT females (∼837 vs. 1043 cm) and decreased incrementally in both groups over time (Figure 6C; white bars). These parallel trajectories were reflected in a repeated measures ANOVA that identified significant main effects of Testing Age and significant main effects of Sex [F_(1,16)_ = 5.81, *p* = 0.028; η^2^ = 0.27] but found no significant or near significant interactions between these two variables. Follow-up pairwise comparisons further showed that sex differences in this measure reached significance for testing when WT rats were 5 months old [t(7) = −2.89, *p* = 0.023, *d* = −1.02, Figure 6C]

At every testing age, male *Pink1^–/–^* rats ambulated at slightly faster speeds and for ∼150–200 cm longer distances than WT males in closed arm spaces (Figures 6B, C; black bars). These differences were consistent with findings from repeated measures ANOVAs. In addition to significant main effects of Testing Age for velocity [F_(2,42)_ = 21.13, *p* < 0.001; η^2^ = 0.50], these comparisons identified significant main effects of Genotype for distance traveled [F_(1,21)_ = 7.90, *p* = 0.010; η^2^ = 0.27]. Follow up pairwise comparisons further that showed that differences in distance traveled among WT and *Pink1^–/–^* males were significant to near significant for testing at all ages [t(7) = −1.47 to −2.67, *p* = 0.016–0.092, *d* = −0.52 to −0.94, Figure 6C]. For female *Pink1^–/–^* rats, average velocities were also faster than WT controls in testing at 3 and 5 months of age (∼11 cm/s, Figure 6B; gray bars). However, the average velocities in this group remained steady at 7, 9 and 12 months of age, thus allowing WT females to “catch up.” The average distances traveled by female *Pink1^–/–^* rats were also roughly ∼100–400 cm longer than those traveled by WT female controls (Figure 6C; gray bars). These group and testing age/repetition-dependent differences were reflected in outcomes from repeated measures ANOVAs. In addition to main effects of Testing Age [F_(3.09–4,58.84–76)_ = 3.82–6.03, *p* = 0.001–0.013; η^2^ = 0.17–0.24], these analyses identified significant interactions between Genotype and Testing Age for velocity [F_(4,76)_ = 2.31, *p* = 0.049; η^2^ = 0.12] and significant main effects of Genotype for average distance traveled [F_(1,19)_ = 11.03, *p* = 0.004; η^2^ = 0.37]. Follow up pairwise comparisons further showed that group/genotype differences were significant for average velocity of ambulation in testing at 3 months of age [t(9) = −3.72, *p* = 0.002, *d* = −1.18; Figure 6B] and for average closed arm distances traveled for testing at 5, 9 and 12 months of age [t(9) = −2.36 to −4.10, *p* = 0.001–0.021, *d* = −0.75 to −1.29, Figure 6C].

### 3.8 Open arm measures

#### 3.8.1 Ambulation

**FIGURE 7:**
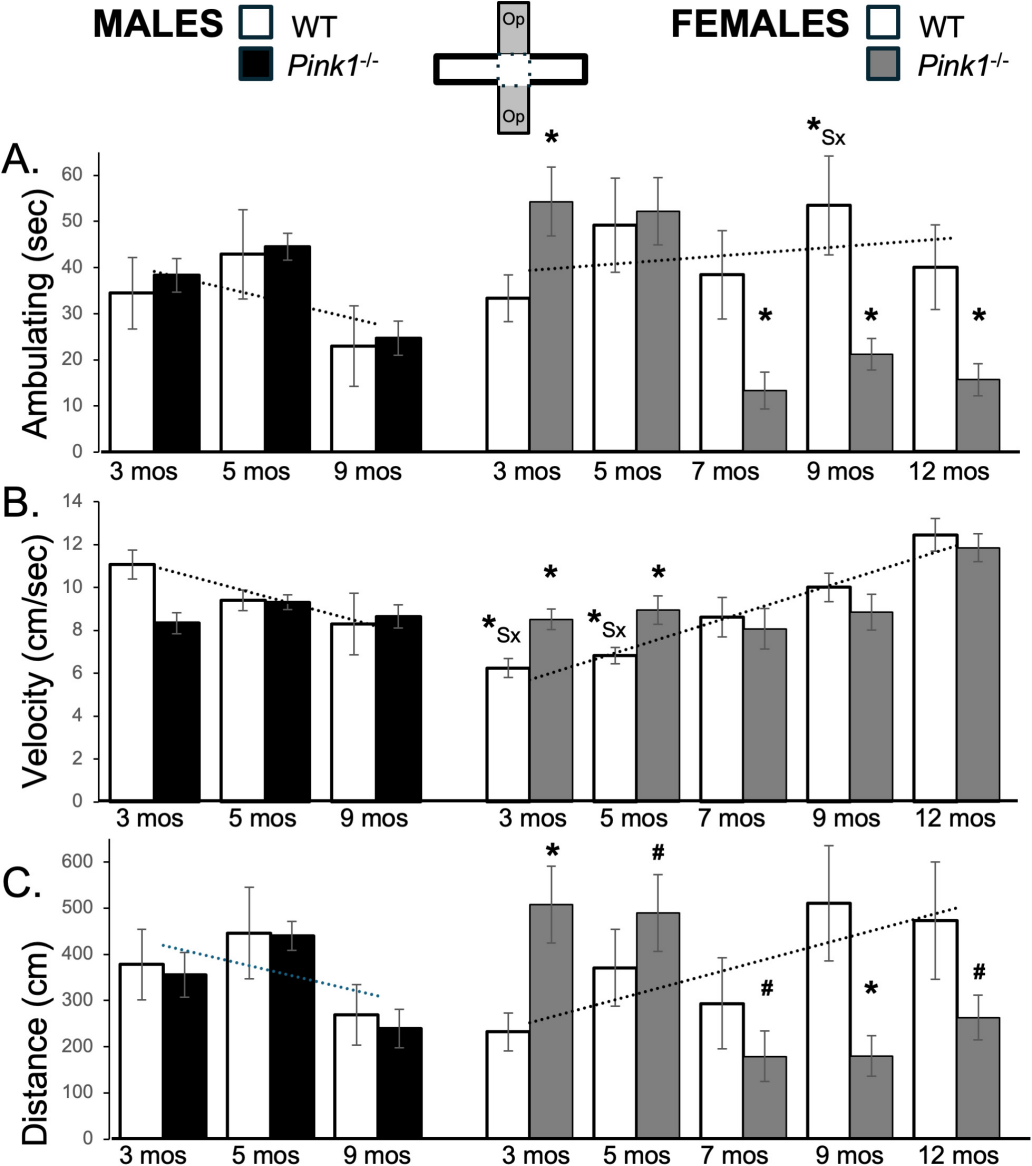
Bar graphs showing average cumulative amounts of time in seconds (s) that rats in each of the four experimental groups spent ambulating **(A)** to time in the open arms of the elevated plus maze (gray zones, inset figure). Average velocity of ambulation [in centimeters/second (cm/s) **B**] and average linear distances traveled in the maze [in centimeters (cm), **C**] over the 5-min trials are also shown. Data from wild type (WT, white bars) and *Pink1^–/–^* (black bars) males, tested at 3, 5 and 9 months (mos) of age are shown in the left column; data from WT (white bars) and *Pink1^–/–^*, gray bars) females, tested at 3, 5, 7, 9 and 12 months of age are shown in the right column. Error bars are standard errors of the mean. For ease of comparison, fitted linear trendlines calculated for the WT groups are shown (dashed lines). Asterisks mark significant differences (*p* < 0.05) within sex between WT and *Pink1^–/–^* rats, hashtags mark near-significant differences (0.05 > *p* < 0.09) within sex between WT and *Pink1^–/–^* rats and asterisks and hashtags superscripted ahead of “Sx” identify data points that are significantly or near significantly different among WT male and female rats.

During initial testing, 3-months-old WT male and WT female rats spent around 33–34 s ambulating in the open arms of the maze (Figure 7A; white bars). Over repeated testing, however, the times that WT males spent ambulating decreased while corresponding in WT females increased. This yielded sex differences in this measure that increased over time. Although a repeated measures ANOVA found no significant main effects of Testing Age or Sex on this variable, it did identify significant interactions between Sex and Testing Age [F_(2,32)_ = 3.47, *p* = 0.043; η^2^ = 0.18]. Follow up pairwise comparisons further showed that sex differences in average ambulating times were significant in testing at 9 months of age [t(7) = −2.33, *p* = 0.05, *d* = −0.82, Figure 7A].

Open arm ambulation times for male *Pink1^–/–^* rats closely matched those of WT controls at all testing time points (Figure 7A; black bars). Thus, repeated measures ANOVAs identified significant main effects of Testing Age [F_(2,42)_ = 10.45, *p* < 0.001; η^2^ = 0.33], but no significant or near significant main effects of Genotype and no significant or near significant interactions between Genotype and Testing Age. In contrast, at 3 months of age, female *Pink1^–/–^* rats spent more time ambulating (∼54 vs. 33 s) compared to WT females (Figure 7A; gray bars). However, in testing at 7, 9 and 12 months of age, the *Pink1^–/–^* females spent less time ambulating (∼13–21 vs. 38–53 s) than controls. These observations were supported first by a repeated measures ANOVA that identified significant main effects of Testing Age [F_(4,64)_ = 7.69, *p* < 0.001; η^2^ = 0.33] and significant interactions between Genotype and Testing Age [F_(4,64)_ = 10.91, *p* < 0.001; η^2^ = 0.41]. Pairwise *post hoc* comparisons further identified significantly more ambulation in the *Pink1^–/–^* cohort at 3 months of age [t(8-9) = −3.25, *p* = 0.005, *d* = −1.03, Figure 7A], and significantly less ambulation in this group in testing at 7, 9 and 12 months of age [t(8-9) = −2.11 to 3.41, *p* = 0.004–0.034, *d* = 0.70–1.08, Figure 7A] compared to WT controls.

#### 3.8.2 Velocity and distance traveled

At 3 months of age, the average speeds of ambulation and total distances traveled within the open arms were greater in WT males than in WT females (Velocity: ∼11 vs. 6 cm/s; Distance ∼380 cm vs. 230 cm, Figures 7B, C; white bars). However, with repeated testing both measures decreased in males and increased in females, thus bringing them closer together toward the end of repeated plus maze testing. These trends were statistically supported. For average distances traveled, a repeated measures ANOVA identified interactions between Testing Age and Sex that approached significance [F_(2,32)_ = 2.92, *p* = 0.068; η^2^ = 0.15]. However, follow up pairwise comparisons showed that sex differences in this measure did not reach significance for testing at any age (Figure 7C). For average velocity of ambulation, a repeated measures ANOVA identified significant interactions between Sex and Testing Age [F_(2,32)_ = 21.58, *p* < 0.001; η^2^ = 0.57] and significant main effects of Sex [F_(1,16)_ = 5.10, *p* = 0.038; η^2^ = 0.24], while follow up pairwise comparisons found significant sex differences in average velocity of ambulation in testing at 3 and 5 months of age [t(7) = 3.88–5.89, *p* = 0.001–0.006, *d* = 1.37–2.08, Figure 7B].

Average speeds of open arm ambulation and average total distances traveled by male *Pink1^–/–^* rats were similar to those of age- and sex matched WT controls (Figures 7B, C; black bars). Accordingly, while repeated measures ANOVAs found significant to near significant main effects of Testing Age for these measures [F_(1.70–2,35,57–42)_ = 3.10–5.46, *p* = 0.008–0.065; η^2^ = 0.13–0.21], no significant or near significant main effects of Genotype were observed and significant interactions between Genotype and Testing Age were only seen for velocity [F_(1.70,35.57)_ = 5.44, *p* = 0.012; η^2^ = 0.21]. Follow up pairwise comparisons further showed that significant group/genotype differences in open arm velocity were limited to testing at 3 months of age [t(7) = 4.23, *p* = 0.002, *d* = 1.50, Figure 7B]. In contrast, open arm ambulation velocity and average total distances traveled by *Pink1^–/–^* female rats were both initially greater than those of WT females (Figures 7B, C; gray bars). However, both transitioned to measures that were similar to lower than those of controls over time. Thus, at 3 months of age, female *Pink1^–/–^* rats) ambulated more quickly (∼8.5 vs. 6.2 cm/s) and covered more distance (∼508 vs. 232 cm) than WT female controls. However, in testing at 7, 9 and 12 months of age, female *Pink1^–/–^* rats ambulated at similar speeds (∼8–11 vs. 8–12 cm/s) while covering less linear distance (∼170–260 vs. 290–500 cm) than the WT females (Figures 7B, C; gray bars). Statistical support for the velocity data included a repeated measures ANOVA that identified significant main effects of Testing Age [F_(3.42,54.65)_ = 16.54, *p* < 0.001; η^2^ = 0.51] and significant interactions between these Genotype and Testing Age [F_3.42,54.65)_ = 4.14, *p* = 0.008; η^2^ = 0.21] and pairwise comparisons showing that group/genotype differences were significant in testing at 3 and 5 months of age [t(9) = −1.97 to −4.34, *p* = 0.001–0.042, *d* = −0.66 to −1.37, Figure 7B]. Similarly, confirmation of the average distance data included, significant interactions between Genotype and Testing Age [F_(4,76)_ = 4.50, *p* < 0.003; η^2^ = 0.19] identified in a repeated measures ANOVA and outcomes from follow up pairwise comparisons showing that distances traveled by the *Pink1^–/–^* group were significantly to near significantly greater than WT in testing at 3 and 5 months of age [t(9) = −1.50 to −3.65, *p* = 0.003–0.084, *d* = −0.47 to −1.15, Figure 7C] and were significantly to near significantly less than WT in testing at 7, 9 and 12 months of age [t(8-9) = 1.53–2.30, *p* = 0.024–0.080, *d* = 0.48 to −0.73, Figure 7C].

### 3.9 Arm subcompartment measures

#### 3.9.1 Closed arms

**FIGURE 8:**
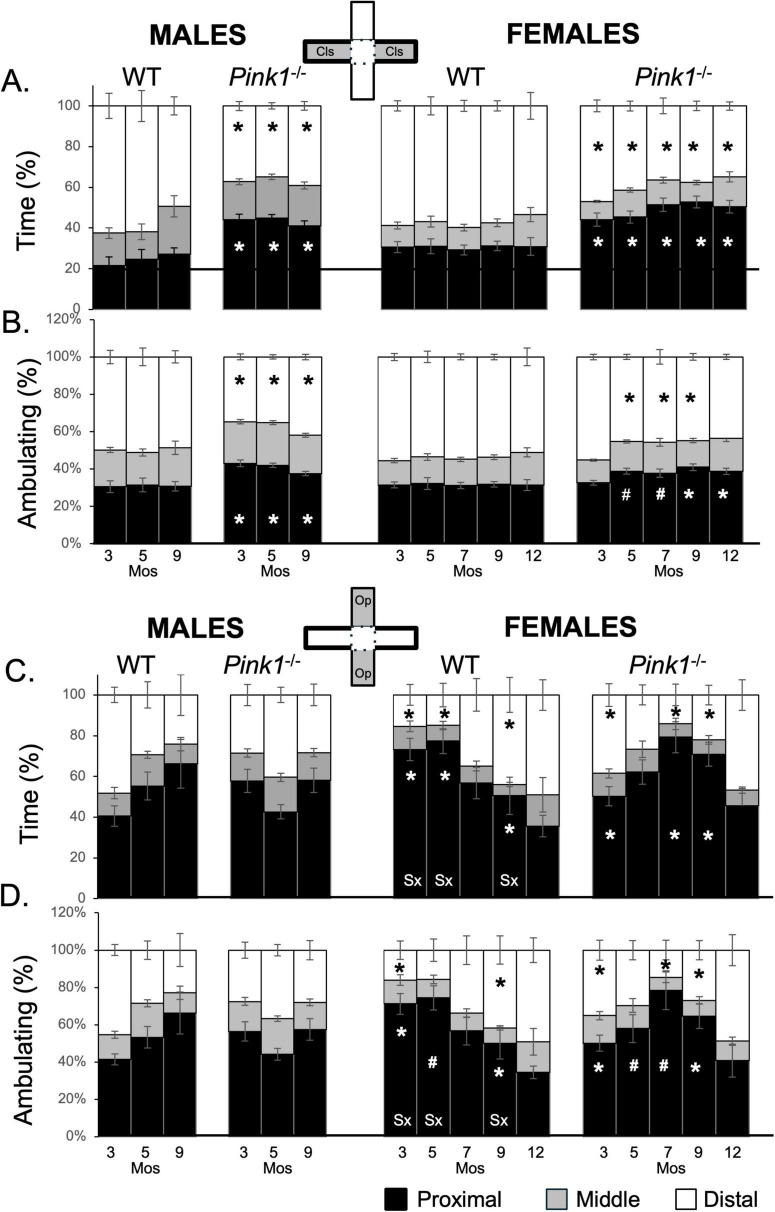
Stacked bar graphs showing average percents (%) of total time **(A,C)** and time spent ambulating **(B,D)** that rats in each of the four experimental groups spent in proximal (black), middle (gray) or distal (white) thirds of closed arms (gray zones, top inset figure; **A,B**) and open arms of the maze (gray zones, bottom inset figure; **C,D**). Data from wild type (WT, white bars) and *Pink1^–/–^* (black bars) males, tested at 3, 5 and 9 months (Mos) of age are shown in the left columns; data from WT (white bars) and *Pink1^–/–^*, gray bars) females, tested at 3, 5, 7, 9 and 12 months of age are shown in the right columns. Asterisks mark significant differences (*p* < 0.05) within sex between WT and *Pink1^–/–^* rats, hashtags mark near-significant differences (0.05 > *p* < 0.09) within sex between WT and *Pink1^–/–^* rats; asterisks and hashtags in bars marked “Sx” at the base identify data points that are significantly or near significantly different among WT male and female rats.

Analyses made with respect to the distal, middle and proximal thirds of the closed arms of the maze showed that WT male and female rats apportioned average total times spent (Figure 8A) and average times spent ambulating (Figure 8B) consistently over time and similarly to one another. Specifically, rats in both WT groups spent roughly 50%–62% of time in these arms in their distal ends (Figure 8A; white bars, first and third columns) and spent about ∼50%–55% of this time spent ambulating (Figures 8B, C; white bars, first and third columns). Both groups also spent relatively little time in middle aspects of the closed arms (Figure 8; gray bars, first and third columns), and spent about ∼23% and 30% of time and ∼17%–30% of times ambulating in the proximal ends of the closed arm spaces (Figures 8A, B; black bars, first and third columns). Repeated measures ANOVAs that compared these values found no significant or near significant main effects of Testing Age or Sex and no significant or near significant interactions between Sex and Testing Age for any of these subcompartment specific measures.

Male and female *Pink1^–/–^* rats also spent least amounts of time in middle portions of the closed arms (Figures 8A, B; gray bars, second and fourth columns). However, rats in both groups spent less time distally and more time in proximal thirds of the closed arms compared to sex- and age-matched WT controls. For male *Pink1^–/–^* rats, 34%–39% of time was spent distally, with 35%–41% of ambulation taking place in these zones (Figures 8A, B; white bars, second column). In contrast, the *Pink1^–/–^* males spent 41%–45% of time and 37%–41% of time ambulating in the proximal ends of the closed arm spaces (Figures 8A, B; black bars, second column). Overall, times spent in the distal thirds of the closed arms were 10%–30% less than those of WT male controls while times spent in proximal thirds of these arms were 7%–20% greater than those of the male controls. Findings for *Pink1^–/–^* females were similar. For these rats, 34%–41% of time and 43%–56% of time ambulating took place in distal aspects of the closed arms (Figures 8A, B; white bars, fourth column) and 42%–53% of time and 31%–40% of time ambulating was spent in the proximal ends of the closed arm spaces (Figures 8A, B; black bars, fourth column). As in males, times spent by female *Pink1^–/–^* rats in distal parts of the closed arms were ∼5%–30% less than corresponding measures in WT females, while times spent in proximal portions of these arms were 15%–30% greater than in WT controls. Repeated measures ANOVAs that compared these distal and proximal measures found no significant or near significant main effects of Testing Age and no significant or near significant interactions between Genotype and Testing Age for either sex. However, significant main effects of Genotype were found for males and females [F_(1,19–21)_ = 15.85–118.24, *p* < 0.001; η^2^ = 0.46–0.86]. Follow up pairwise comparisons of the data for males showed that the times spent by *Pink1^–/–^* males were significantly greater than WTs for proximal arms [t(7) = −2.42 to −3.221, *p* = 0.007–0.023, *d* = −0.85 to −1.14, Figures 8A, B] and significantly less than WT for distal parts of closed arm spaces times [t(7) = 1.93 to −3.08, *p* = 0.009–0.047, *d* = 0.68–1.09, Figures 8A, B; second column]. Corresponding analyses for female rats showed that most measures were significantly or near significantly different among *Pink1^–/–^* and WT female controls [t(10) = −1.65 to −4.76, *p* = 0.001–0.066, *d* = −0.52 to −1.50, Figures 8A, B; fourth column). The exceptions were data collected at 3 months of age and measures of time spent ambulating in distal arms at 12 months of age.

#### 3.9.2 Open arms

Rats in all groups spent minimal time in middle portions of open arms of the maze (Figures 8C, D; gray bars). However, their apportionment of times spent in distal and proximal parts of the open arms differed across groups and over time. For example, WTs males initially spent proportionally more time in distal compared to proximal ends of the open arms (Total time spent = ∼50 vs. 44%; Time spent ambulating = ∼45 vs. 41%, Figures 8C, D; white vs. black bars, first column). However, by 9 months of age, greater percentages of time were being spent proximally rather than distally (Total time spent = 65 vs. 25%; Time spent ambulating = ∼66 vs. 23%, Figures 8C, D; white vs. black bars, first column). Wild type females, on the other hand, initially spent greatest percentages of time in proximal rather than distal thirds of the open arms (Total time spent = ∼73 vs. 15; Time spent ambulating = ∼71 vs. 16%). However, by 9 months of age, there rats were spending similar amounts of times distally and proximally (Total time spent = ∼44 vs. 50%; Time spent ambulating = ∼42 vs. 50%) and by 12 months of age they were spending more time in distal compared to proximal ends of the open arms (Total time spent, Time spent ambulating = ∼50 vs. 35%, Figures 8C, D; white vs. black bars, third column). These dynamics resulted in sex differences in open arm occupancies that peaked in early testing and diminished at intermediate and later testing ages. Thus, repeated measures ANOVAs that compared total and ambulation times in WT males and females at 3, 5 and 9 months of age found no significant or near significant main effects of Testing Age or Sex but did find significant interactions between Testing Age and Sex for both measures [F_(2,32)_ = 6.00–15.02, *p* = 0.001–0.006; η^2^ = 0.27–0.48]. Follow up pairwise comparisons further showed that sex differences were significant to near significant for nearly all measures in testing at 3, 5 and 9 months of age [t(7) = −2.31 to 6.75, *p* = 0.001–0.055, *d* = −0.82 to 2.39, Figures 8C, D, third column]. The single exception was ambulation times for distal arms at 5 months of age.

In contrast to WT controls, male *Pink1^–/–^* rats consistently spent more than 50% of total time and total time ambulating in proximal parts of the open arms (Figures 8C, D; black bars, second column), 5%–15% of time in middle thirds (Figures 8C, D; gray bars, second column) and approximately 30% in the distal ends of these open spaces (Figures 8C, D; white bars, second column). Although there were some differences in testing at 3 months of age, at 5 and 9 months apportionments of time were highly similar in *Pink1^–/–^* and WT males. Repeated measures ANOVAs that compared total and ambulation times in open arms among *Pink1^–/–^* and WT males identified no main effects of Genotype and no significant interactions between Testing Age and Genotype.

The percentages of time that female *Pink1^–/–^* rats spent and spent ambulating in subcompartments of open arms uniquely followed inverted “U” shaped patterns (Figures 8C, D, fourth column). Thus, *Pink1^–/–^* females rats started out spending more slightly more time in proximal compared to distal ends of the open arms (Total time spent = ∼49 vs. 39%; Time spent ambulating = ∼47 vs. 37%) but over the next few testing sessions, they spent more time spent in proximal compared to distal arm subcompartments (Total time spent = ∼87 vs. 8%; Time spent ambulating = ∼82 vs. 11%). However, in testing at 9 and 12 months of age, these patterns reversed, as *Pink1^–/–^* rats began spending more times distally, reverting to values that were similar to those observed during testing at 3 months of age. These dynamics were reflected in repeated measures ANOVAs that found no significant or near significant main effects of Genotype, but did identify significant main effects of Testing Age [F_(4,76)_ = 3.92–9.28, *p* = 0.001–0.006; η^2^ = 0.17–0.37] and significant interactions between Genotype and Testing Age for both measures [F_(4,76)_ = 4.05–7.44, *p* = 0.001–0.009; η^2^ = 0.16–0.32]. Follow up pairwise comparisons further showed that group/genotype differences in total times spent were significant for proximal and distal arms in testing at 3, 7 and 9 months of age [t(9) = −2.19 to −4.24, *p* = 0.001–0.028, *d* = −0.69 to 1.41, Figures 8C, D; fourth column] and that differences in ambulation were significant to near significant in these zones in testing at 3, 5, 7 and 9 months of age [t(7) = −1.55 to 3.75, *p* = 0.002–0.078, *d* = −0.49 to 1.19, Figures 8C, D; fourth column].

## 4 Discussion

Anxiety disturbances are commonly occurring non-motor symptoms in PD that negatively impact patients’ lives, bring greater responsibilities to caregivers and pose challenges to health care providers in terms of making accurate diagnoses and providing safe and effective symptom relief (Blundell et al., 2023; Khatri et al., 2020; Ray and Agarwal, 2020; Weintraub, 2020). Because signs of anxiety predominate among female patients (Dissanayaka et al., 2014; Zhu et al., 2017)– the biological sex that is less vulnerable to PD overall, information about the neurobiological underpinnings and optimal treatments strategies for these non-motor signs have been difficult to ascertain from clinical studies alone. Further, while impacts on anxiety have been identified in a number of preclinical models of PD, for many these affective changes are either similar across biological sex, predominant in males or occur in opposite direction of that is observed clinically (Campos et al., 2013; Faivre et al., 2019; Wichmann et al., 2025). The studies presented here tested the hypothesis that *Pink1^–/–^* rats more aptly recapitulate core clinical characteristics of anxiety disturbances in PD, including their early premotor/prodromal onset and increased prevalence and severity in females. These predictions were largely borne out in longitudinal elevated plus maze testing that first identified dynamic, sex-specific behavioral profiles in WT male and female rats. Specifically, WT male rats were found to initially explore all parts of the plus maze and especially its open arms more so than WT females. However, with repeated testing WT males incrementally reduced exploration and spent less time in open arms and more time in the closed arm spaces. Wild type female rats, on the other hand, overcame initial caution and engaged in progressively more ambulation and greater exploration of open relative to closed arm compartments over time. These data served as age-and sex-matched controls in defining the effects of *Pink1* gene knockout on elevated plus maze performance in male and female *Pink1^–/–^* rats. These comparisons showed that male *Pink1^–/–^* rats consistently displayed hyperlocomotion in the center platform and closed arms of the maze; at every age evaluated, male *Pink1^–/–^* rats made more entries into these spaces, spent more time ambulating within them and covered greater distances relative to WT male controls. They also spent more time in the proximal and less time in the distal thirds of the closed arm compartments. In contrast, there were minimal difference between *Pink1^–/–^* and WT males in entries or occupancies of open maze arms and minimal difference in any behavioral measure in the *Pink1^–/–^* male group over time. Overall, male *Pink1^–/–^* rats exhibited greater activity, little to no indications of increased anxiety and less habituation to repeated testing than sex-matched controls. During testing at 3 and 5 months of age, female *Pink1^–/–^* rats also showed hyperlocomotion. However, in addition to making more entries into the maze’s center and closed arms, the *Pink1^–/–^* females also showed increased entries, increased ambulation and greater distances traveled in open arms compared to female controls. They also spent proportionally more time in the distal thirds of these open spaces competed to WTs. Thus, while similar to *Pink1^–/–^* males in showing hyperlocomotion, the young adult *Pink1^–/–^* female rats displayed multiple behaviors suggesting anxiety relative to WT controls rather than unchanged behavioral indicated of anxiety observed in *Pink1^–/–^* males. The EPM behaviors of *Pink1^–/–^* female rats changed abruptly however, in testing at all subsequent ages. First, at 7, 9 and 12 months of age, *Pink1^–/–^* females spent more and more time in closed arms and less time in the maze’s center and open arms. In fact more than half of the *Pink1^–/–^* female cohort made no entries at all into the open arms and among those that did, they rarely explored these open spaces beyond their proximal thirds. These increased/increasing signs of anxiety were in sharp contrast to behaviors in WT females that explored the open spaces of the maze more and more over time, and clearly demonstrate the development of an anxiety phenotype in *Pink1^–/–^* females that is largely absent in male *Pink1^–/–^* rats. The strengths, limitations and translational implications of these findings are discussed further below, following a brief consideration of the extant literature describing sex differences in behavioral measures of anxiety in wild type rodents.

### 4.1 Sex differences in elevated plus maze performance in rats

Sex differences have been described in rats and mice in several classical behavioral indices of anxiety, including those observed in Novel Open Field, Light/Dark Box and Elevated Plus Maze testing (Kokras and Dalla, 2014; Palanza, 2001; Wegener and Neigh, 2021). Although, the data are not always consistent across studies and can be even more variable across tasks, for EPM testing most studies report either no sex (Fabris et al., 2022; Tucker and McCabe, 2017) or increased indices of anxiety in males (Knight et al., 2021; Ou et al., 2019; Scholl et al., 2019; Xiang et al., 2011). Because most of these studies examined single time points, they may be best compared to the earliest time points evaluated in WT male and female rats here where during initial testing, WT females engaged in more locomotion, spent more time and made more entries in closed arms and spent less time and made fewer entries into open arms and open center platform of the maze than WT males. The WT females also ambulated faster and covered greater distances in closed arms, and ambulated more slowly and covered less ground than males in the open arms and center platform. Finally, WT rats of both sexes showed a preference for the distal thirds of the closed maze arms. However, WT males spent proportionately more time in distal compared to proximal parts of the open arms, while females largely avoided the distal ends of these open spaces. Overall, these data suggest initially greater behavioral expressions of anxiety in WT females compared to males which is not unprecedented, but is also not the typical pattern for sex differences- or lack thereof, most often observed in EPM testing in male and female rodents (Börchers et al., 2022).

It is important to emphasize that the present study was not optimally configured to evaluate quantitively sex differences in the data, as the male and female examined were purchased and behaviorally tested separately– roughly one year apart. In addition, sex differences in early handling histories related to determinations of estrous cycle regularity in WT females might also confound direct, quantitative comparisons of behavioral EPM measures in WT males and females. However, qualitative differences were also noted in the ways that WT male and female rats adapted to repeated plus maze testing over time that are less likely to be impacted by these conditions. Specifically, data from whole maze, compartment- and subcompartment-specific analyses alike all showed that over the course of repeated testing, WT males generally slowed down, covered less ground and spent more time in enclosed rather than open parts of the maze. Wild type females, on the other hand, progressively increased ambulation, ambulation velocity and distances traveled, particularly within open parts of the maze including the distal ends of the open arms. These dynamic behavioral changes in females are similar to those previously observed in WT Long Evans in rats tested bimonthly from 2 to 8 months of age (Marquis et al., 2020). On the other hand, the decrementing behavioral metrics noted in the WT males are not well aligned with previous reports of behavioral changes in EPM testing following more U-shaped curves in male rats that were repeatedly evaluated at 4, 8 and 12 months of age (Hoffmeister et al., 2022).

In all three studies, it is notable that the months-long intertrial intervals uses were beyond timeframes associated with “one-trial tolerance.” This term refers to a an increase in anxiolytic-insensitive measures of anxiety, e.g., reduced open arm occupancy, that is seen in animals that are re-tested on the EPM within 14 days (File et al., 1990) but is absent when intertrial intervals are extended to at least 28 days and when changes are made to distal testing room cues (Schneider et al., 2011). Both of the latter two conditions apply to the present studies. Thus, observations in WT female rats of increasing ambulation and occupancies of the most unprotected, open parts of the maze are provisionally interpreted as signs of habituating to repeated testing and waning anxiety. The incremental decreases in maze exploration overall and the increased times spent in closed relative to open maze spaces observed in WT male rats, on the other hand, could reflect either sensitization to testing and increasing anxiety or diminished motivation to explore as the novelty of testing wears off. Anxiolytic/anxiogenic drug challenges may be especially useful in more definitively identifying the affective bases for evolving EPM behaviors in WT rats of both sexes. What is clear in the meantime, however, is that there are striking, sex-specific temporal dynamics for most behavioral metrics evaluated in this study. The impacts of the *Pink1^–/–^* genotype on EPM performance– that were defined in this study with respect to these age- and sex-specific baselines, are considered further below.

### 4.2 Comparisons to previous EPM studies in *Pink1^–/–^* rats

#### 4.2.1 Males

There are two previous studies that compared EPM performance in male *Pink1^–/–^* and WT rats. In one, rats were tested at a single timepoint when subjects were 6–8 months of age (Cai et al., 2019). These studies showed that *Pink1^–/–^* male rats spent less time and made fewer entries into open arms and into maze arms in general compared to WT male controls (Cai et al., 2019). These indices of relative open arm avoidance data suggested elevated anxiety in male *Pink1^–/–^* rats. In the second EPM study, rats were longitudinally tested at 4, 8 and 12 months of age (Hoffmeister et al., 2022). Data from this study suggested that the phenotype of elevated anxiety in male *Pink1^–/–^* rats is transient. Due to substantial number of rats from both groups failing to enter open arms, behavioral measures evaluated were closed arm entries, times spent in closed arms and calculated ratios of closed arm preference. No significant differences were found for any of these measures among *Pink1^–/–^* and WT control males in testing at 4 and 12 months of age (Hoffmeister et al., 2022). However, in testing at 8 months of age, the numbers of closed arm entries, times spent in closed arms and calculated closed arm preference ratios were all greater in *Pink1^–/–^* compared to WT males (Hoffmeister et al., 2022). This bias toward closed, relatively protected parts of the maze complements open arm findings from the prior study could indicate a phasic elevation in anxiety in male *Pink1^–/–^* rats at around this age. However, data from the present study also showed that *Pink1^–/–^* male rats more entries, spent more time ambulating and traveled for longer distances in closed arms compared to WT males, but did not show differences from controls for any open arm measurements. Thus, the data from this study argue that hyperlocomotion may be the principal driver of behavioral differences noted in EPM performance in *Pink1^–/–^* compared to WT male rats. These conclusions are similar to those drawn from Novel Open Field testing which has shown that male *Pink1^–/–^* rats exhibit (Dave et al., 2014) but spend similar amounts of time in open arena centers as WT controls (Lechner et al., 2022; Lechner et al., 2025). Future studies are needed to determine whether and to what extent differences observed across studies using EPM testing may have been influenced by study-to-study differences in testing conditions, including testing rats during subjective days vs. nights and the use of dim, red vs. ambient white room lighting.

#### 4.2.2 Females

One previous study has examined performance of female *Pink1^–/–^* rats in EPM testing. In this study, rats were longitudinally tested on EPM at 2, 4, 6 and 8 months of age and were also tested on the Light/Dark Box paradigm at 3, 5 and 7 months of age (Marquis et al., 2020). During EPM testing at 2, 4 and 6 months old, female *Pink1^–/–^* rats were found to consistently make more open arm entries and spend longer periods of time in open arms than WT females (Marquis et al., 2020). In Light/Dark box testing at 3, 5 and 7 months of age, the same female *Pink1^–/–^* rats were also shown to spend less time than WT controls in the darkened portion of the apparatus (Marquis et al., 2020). Thus, outcomes from both paradigms (Hoffmeister et al., 2022) suggest that anxiety levels are reduced in young adult female *Pink1^–/–^* rats relative to WT female controls (Marquis et al., 2020). This phenotype is further corroborated in a Novel Open Field study that showed that at 2-months of *Pink1^–/–^* female rats spent significantly more time in the open, unprotected space of the arena center than WT controls (Lechner et al., 2022). However, in EPM testing at 8 months old, indices of anxiety in *Pink1^–/–^* females changed dramatically. Specifically, at this age female *Pink1^–/–^* rats made markedly fewer entries and spent noticeably less time in open arms compared to WT females (Marquis et al., 2020)–both of which are behavioral measures suggesting increased anxiety in the *Pink1^–/–^* group relative to controls. A similar reversal was observed in the female *Pink1^–/–^* rats evaluated here. Thus, in testing at 3 and 5 months of age multiple measures of behavior indicated that levels of anxiety in the *Pink1^–/–^* females were significantly lower than those of WT female controls. For example, at these ages *Pink1^–/–^* females made significantly more open arm entries, stayed for significantly longer in these open spaces and spent significantly more time in distal ends of these unprotected spaces longer periods of time than control females. However, in testing at 7, 9 and 12 months of age, a very different set of behaviors emerged that suggested that anxiety levels had become significantly higher in *Pink1^–/–^* compared to WT female controls. The present studies thus confirm and extend evidence for a phenotype of lower-than-normal levels of anxiety in young adult female *Pink1^–/–^* rats (3–6 months), a striking shift in this phenotype to one characterized abnormally high levels of anxiety in adulthood (7–8 months) and the persistence in this heightened anxiety state through at least 12 months of age. It is notable that these largely consonant findings were obtained despite study-to-study differences in the times of day that rats were tested and the lighting conditions under which testing occurred.

### 4.3 Study strengths, limitations and translational implications

As has been previously reviewed, the advantages of genetic rodent models for translational studies of PD include the spontaneous onset and progression of pathophysiological processes and in several cases, the emergence of non-motor behavioral deficits that precede the appearance of motor impairments (Creed and Goldberg, 2018). In several of these models, anxiety phenotypes have been observed (Campos et al., 2013; Decourt et al., 2021; Dovonou et al., 2023). However, models in which *PINK1/Pink1* function have been perturbed can be predicted to be of especial interest. First, *PINK1/Pink1* has been shown to play important roles in mediating and regulating stress-induced mitophagy (Bader and Winklhofer, 2020). Further, impaired mitophagy induced by diminished, *PINK1/Pink1* function has also been shown to exacerbate cellular stress responses (Grigoruţă et al., 2020) and to increase behavioral expressions of stress and anxiety (Johnson and Li, 2022; Li et al., 2024). Finally, and most directly relevant to PD are findings showing that among patients with monogenic forms of illness, those in whom disease is related to *PINK1* mutations are at elevated risk for neuropsychiatric symptoms, including anxiety (Ephraty et al., 2007; Kalinderi et al., 2024). Thus, it is perhaps not surprising that evidence for anxiety phenotypes in *Pink1* deficient and *Pink1^–/–^* rats and mice continues to grow.

As described above, the present study used EPM testing to further explore questions about the development of behavioral indices of anxiety in male and female *Pink1^–/–^* rats. Although a limitation of this approach is the use of a single paradigm to measure and make inference about anxiety, this was mitigated in part by analytical strategies that allowed some indices of anxiety to be cross-confirmed. For example, in addition to increased open arm entries and occupancies, supporting evidence for reduced anxiety in *Pink1^–/–^* females during young adulthood (3 and 5 months of age) also included faster and farther ambulation within these arms and increased exploration out to their most distal, most exposed ends relative to controls. The present findings also gain support by aligning with previous studies were other behavioral measures of anxiety were evaluated (above). Thus, the present studies not only add to a growing consensus for *Pink1^–/–^* rats as modeling anxiety disturbances in PD, they also affirm the relatively unique features of this strain in modeling the sex differences that distinguish symptoms of anxiety in PD. These characteristics make *Pink1^–/–^* rats attractive models for testing novel treatments and filling other gaps in knowledge in ways that minimize social/societal factors including the stigma associated with mental illness that challenge clinical investigations of anxiety in PD. By advancing understanding of the neurobiological underpinnings of these signs, these models may also be useful in helping patients recognize anxiety disturbances as integral parts of a neurodegenerative disease. This in turn could be useful in reducing what are alarming rates of patient reticence to disclose symptoms to health care providers and seek medical treatments for these debilitating signs (Prediger et al., 2012). Finally, findings with respect to EPM performance specifically identify potential strengths of *Pink1^–/–^* rat strains for translational investigation of the imbalances in approach/avoidance conflict that are often seen in PD patients. While generally characterized as decreased novelty seeking and increased harm avoidance– even under conditions of relative safety (Costa and Caltagirone, 2015; Sheynin et al., 2020), these impacts could be related to the sex differences that have been described in patients’ risk vs. rewards assessment of therapeutic deep brain stimulation (DBS). Specifically, female patients with PD have been described as being more fearful of adverse outcomes, less trusting of medical advice and to wait longer and require more social and emotional support in order to go through with the invasive surgical procedure (Fullard et al., 2025). Thus, vulnerability to maladaptive risk aversion could account for women being less likely overall to receive DBS (Hariz and Hariz, 2000; Hariz et al., 2013). The EPM analyses carried out here suggest that sex-specific disturbances in approach/avoidance conflict may also characterize *Pink1^–/–^* rats and model those that interfere with clinical care in PD. Specifically, this study showed that not only did female *Pink1^–/–^* rats develop exaggerated avoidance of open maze spaces, they also spent greater amounts of time than WT controls at proximal ends of the maze’s closed arms. Thus, rather than seeking protections of the distal ends of these enclosed spaces, these rats hovered at a decision point in the maze, albeit from the safe vantage point of closed arm. Perhaps like the greater willingness of male PD patients to proceed with relatively risky DBS procedures (Fullard et al., 2025), male *Pink1^–/–^* rats not only showed no heightened aversion to open arms, they also spent more time weighing risks and rewards of entering the open or close maze arms from the highly vulnerable spot of the open maze center. Thus, although further studies are needed, in addition to supporting an anxiety phenotype in *Pink1^–/–^* rats in general, the use of behavioral measures beyond those that are standard in EPM testing may have uncovered impacts of the *Pink1^–/–^* genotype on approach/avoidance endpoints of anxiety that are understudied in PD but that if better understood and treated, could improve motor as well as non-motor outcomes for PD patients of both sexes.

### 4.4 Further relationships to sex-specific deficits in cognition and memory in *Pink1^–/–^* and other rat models of PD

The longitudinal strategy used in this study revealed systematic changes in behavioral EPM measures in WT male and female rats and more abrupt shifts in performance among female *Pink1^–/–^* rats across the span of repeated testing. This suggests that rats in these groups retained some form of memory for prior testing experiences and used this information to update subsequent maze performances, albeit in different, group-specific ways. In contrast, male *Pink1^–/–^* rats showed almost no appreciable change in behavioral EPM measures over time. This suggests that these rats had limited access to memories for past testing experiences which is consistent with recent data showing that male but not female *Pink1^–/–^* rats have developed significant, enduring deficits in episodic memory before 3 months of age (Desai et al., 2025). Specifically, longitudinal testing using the What Where When Episodic-like Memory task (WWW) in male and female WT and *Pink1^–/–^* rats showed that all three memory domains are profoundly impaired in *Pink1^–/–^* males from 3 months of age on, whereas WWW performance in *Pink1^–/–^* females remained largely intact until testing at 12 months of age (Desai et al., 2025; Desai et al., 2025). It was further noted that male *Pink1^–/–^* rats consistently spent more time in the center platform of the maze than any other group. Although this part of the maze is open, this measure is rarely included in EPM studies and is generally not viewed as an index of anxiety. However, situated at the entry point of the four maze arms, the extended times spent in this part of the maze could signal deficits in response selection or decision making, similar to those that are seen in PD patients (Rutledge et al., 2009; Ryterska et al., 2013; Sinha et al., 2013). In fact, male *Pink1^–/–^* rats have been shown to struggle at decision points in navigating a complex spatial maze (Soto et al., 2024b). Although it is currently unknown whether *Pink1^–/–^* females are also impaired in spatial navigation tasks, it has been shown that while male *Pink1^–/–^* rats develop progressive deficits in a battery of object recognition-based memory paradigms (Novel Object Recognition, Novel Object Location, Object-in-Place tasks) at around 5 months of age (Desai et al., 2025; Desai et al., 2025; Pinizzotto et al., 2022), female *Pink1^–/–^* rats show no deficits in any of these object recognition based memory tasks in testing extended from 3 through 12 months of age (Desai et al., 2025; Desai et al., 2025). Together with the present data, findings obtained to date with respect to cognition and memory suggest two things. First, that in addition to construct validity and face validity for a range of motor deficits, that the *Pink1^–/–^* rat of PD model also recapitulates clinical sex differences for non-motor deficits involving cognition and memory where males are more at risk, and for non-motor deficits affecting anxiety where female PD patients are more vulnerable. Further, the emergence of non-motor deficits within sex and their divergence across sex seem to occur during a similar time window of between 5 and 7 months of age. As considered below, this identifies a surprisingly narrow timeframe over which impacts of loss of *Pink1* function in the male and female brain appear to produce very different, clinically relevant behavioral deficits in these animal models.

## 5 Summary, conclusions and future directions

Over the course of illness, as many as 49% of patients diagnosed with PD will experience some form of anxiety disturbance. The negative consequences of these non-motor symptoms for patients and caregivers are amplified by the fact that safe and effective treatments for them are often lacking. Resolving the neural bases of these symptoms and developing better ways to treat them are needed and may be especially important for female patients, who are more often and/or more severely affected by signs of anxiety in PD. This progress can be facilitated by preclinical animal models that not only develop anxiety but do so in ways that recapitulate the clinical sex differences in these disturbances. The data presented here identify *Pink1^–/–^* rats as one of a very few animal models of PD that meet these criteria. Thus, male and female *Pink1^–/–^* rats initially show hyperlocomotion and broad, possibly impulsive exploration of all portions of the elevated plus maze, including its open, relatively unprotected spaces. However, by 7 months of age, behaviors in female *Pink1^–/–^* rats–and only the females, evolved into ones that continue to show signs of hyperactivity but that also reflect significantly heightened anxiety, e.g., avoidance of open arms and especially their distal ends. Thus, while a surprising number of animal models of anxiety in disease states including PD fail to emulate clinically observed sex differences, the data presented here add to a growing list of the ways in which *Pink1^–/–^* rats model sex differences in prevalence, timing and severity that characterize major motor and non-motor deficits in PD. A limitation of the present study was the lack of biological measurements in animal subjects. However, the data do identify a well-defined age interval over which non-motor deficits in cognition and affect sex-specifically emerge and diverge in this strain. This temporal framework can be used to guide both retrospective analyses of the literature and future studies with common goals of identifying the circuits, cells and/or molecular processes that are affected by loss of function *PINK1/Pink1* mutations/manipulations in the male and female brain and that may be responsible for the very different behavioral profiles seen clinically and in *Pink1^–/–^* rats. This information in turn should be useful in informing clinical, translational and drug discovery studies leading to the development of better able to mitigate disturbances particularly in non-motor domains including anxiety, where symptoms are often treatment resistant. Further, with data in hand showing that *Pink1^–/–^* rats sex-specifically model motor, cognitive and affective complications of PD, this strain may offer an especially powerful platform for learning more about the predictive powers and interactions among major PD symptom clusters, and for making therapeutic advances that avoid common complications of treatments that benefit motor function exacerbating non-motor deficits and vice versa (Rektorova, 2019).

## Data Availability

The datasets presented in this study can be found in online repositories. The names of the repository/repositories and accession number(s) can be found below: https://osf.io/syhka/?view_only=59ce326df9a04787984195e8ac7c5252.
